# Biosynthesis of bimetallic silver–copper oxide nanoparticles using endophytic *Clonostachys rosea* ZMS36 and their biomedical applications

**DOI:** 10.3389/fmicb.2025.1581486

**Published:** 2025-05-09

**Authors:** Zhijiang Chen, Tianyu Lv, Yuxing Zhang, Weitao Kong, Xixian Li, Siyun Xie, Jiaqi Li, Yu Long, Liqing Chen, Jiarong Liu, Zhiqi Li, Xingda Zeng, Zujun Deng

**Affiliations:** Guangdong Provincial Key Laboratory of Pharmaceutical Bioactive Substances, School of Basic Medical Sciences, Guangdong Pharmaceutical University, Guangzhou, China

**Keywords:** endophytic fungi, medicinal plant, Ag-CuO NPs, antibacterial agents, anticancer agents

## Abstract

Bimetallic nanoparticles (BMNPs) have garnered significant interest owing to their exceptional physicochemical properties. However, there have been few reports of the biosynthesis of BMNPs using endophytic fungi from medicinal plants. The objectives of this study were to isolate endophytic fungi from medicinal plant *Anemarrhena asphodeloides* to synthesize bimetallic Ag-CuO nanoparticles (Ag-CuO NPs), characterize the biosynthesized Ag-CuO NPs and assess their bioactivity and biosafety. The endophytic fungus ZMS36 capable of biosynthesizing Ag-CuO NPs was isolated from medicinal plant *A. asphodeloides* and identified as *Clonostachys rosea*. The Ag-CuO NPs were biosynthesized using endophytic *C. rosea* ZMS36 and characterized by UV-visible, SEM, TEM, EDS, XRD, and FTIR. The Ag-CuO NPs exhibited good antibacterial activity against *Pseudomonas aeruginosa, Staphylococcus aureus, Escherichia coli, Staphylococcus epidermidis, Salmonella typhimurium*, and *Shigella dysenteriae*. They also significantly inhibited the growth of MRSA and the expression of *mecA* gene, especially in conjunction with vancomycin, the preferred antibiotic for clinical treatment of MRSA infections. The Ag-CuO NPs showed promising anticancer activity in antiproliferative assays on the tumor cell lines HeLa, PDSF, and A549. Furthermore, the Ag-CuO NPs inhibited the migration of HeLa cells as well as angiogenesis in chicken embryos, helping to inhibit tumor metastasis. Interestingly, the Ag-CuO NPs showed low cytotoxicity, indicating good biocompatibility. This study revealed the potential of endophytic fungi from medicinal plants to synthesize BMNPs and highlighted biosynthetic Ag-CuO NPs as promising novel antibacterial and anticancer nanodrugs for future biomedical applications.

## 1 Introduction

Metallic nanoparticles (MNPs) have attracted significant interest from researchers because of their promising applications in biomedicine, biopesticide, electronics, optics, and catalysis, which are attributed to their unique physical and chemical properties (Jiang et al., 2020; Jamil et al., 2024). In most cases, bimetallic nanoparticles (BMNPs) typically exhibit more intriguing properties including optical, magnetic, electronic, catalytic, and antioxidant properties compared to the monometallic nanoparticles (MMNPs) due to the synergistic properties between the two different metal parts (Arora et al., 2020). Additionally, they can also show some novel properties and capabilities due to synergistic effects (Kumari et al., 2015; Zhang et al., 2011). Thus, BMNPs have recently attracted widespread attention. The Au-Ag nanoparticles (NPs) synthesized from the root extract of *Asparagus racemosus* exhibited excellent antibacterial potential (Amina et al., 2020). Au-Pt, Cu-Pt, and Ag-Cu NPs have also demonstrated strong antimicrobial properties (Dobrucka and Dlugaszewska, 2018; Fan et al., 2021; Zhao et al., 2014). Ag-Cu and Cu-Zn NPs were found to be capable of disrupting the metabolism of cancer cells (Merugu et al., 2021). Riaz et al. (2020) reported that Ag-Ni and Ag-Cu NPs exhibited strong antioxidant activity in DPPH free radical scavenging and hydroxyl radical scavenging assays.

Currently, the synthesis of BMNPs still predominantly relies on chemical and physical processes, such as laser ablation, milling, or the addition of chemical reducing agents to metal ion solutions. However, these methods are energy intensive, time consuming and often involve the use of toxic chemicals that produce hazardous byproducts (Loza et al., 2020; Letchumanan et al., 2021). In contrast, biosynthesis offers a more environmentally friendly and safer alternative for BMNPs synthesis (Hosseini and Sarvi, 2015; Sharma et al., 2019). The biosynthesis of metallic NP involves a variety of microorganisms and plants, including bacteria, fungi, yeast, algae, actinomycetes, plants, and phytochemicals (Sharma et al., 2019; Prabakaran and Rajan, 2021; Singh et al., 2016). Among them, the microbial synthesis of NPs offers distinct advantages over plant-mediated synthesis, primarily due to the ease of cultivating and reproducing microbes (Velsankar et al., 2020). Numerous studies have demonstrated that fungi are capable of synthesizing MNPs through the production of various enzymes, such as α-NADPH-dependent reductases, nitrate-dependent reductases, and the extracellular shuttle quinone (Anil Kumar et al., 2007; Deepa and Panda, 2014; Durán et al., 2005; Peng et al., 2024). Medicinal plants are important sources of bioactive natural compounds, showcasing remarkable pharmacological and therapeutic properties (Luo et al., 2024; Zeng et al., 2020). Endophytic fungi of medicinal plants are particularly noteworthy, as they produce a number of natural bioactive secondary metabolites that are identical to those produced by their host plants (Gupta et al., 2020). These enzymes and pharmacologically active secondary metabolites not only contribute to the formation of NPs, but can also be attached to the synthesized metallic NPs, thereby providing additional benefits that increase the efficacy of metallic NPs (Rahman et al., 2019). Additionally, enzymes and pharmacologically active secondary metabolites function as capping agents, preventing the aggregation of the NPs and the formation of long-lasting, stable structures (Anil Kumar et al., 2007; Alghuthaymi et al., 2015). Therefore, endophytic fungi from medicinal plants are an ideal resource for synthesizing BMNPs. Several MMNPS such as AgNPs synthesized using endophytic fungi isolated from medicinal plants showed significant antimicrobial efficacy (Devi et al., 2014; Mohamed et al., 2019). The CuO NPs synthesized by the endophytic fungus *Aspergillus terreus* FCBY1 had been demonstrated to exhibit good antimicrobial, antioxidant, and anticancer activities (Mani et al., 2021). ZnO NPs synthesized by the endophytic fungus *Aspergillus niger* exhibited excellent *in vitro* and *in vivo* antimicrobial activity against *Staphylococcus aureus* (Abdelkader et al., 2022). However, to date, research on the synthesis of BMNPs using endophytic fungi of medicinal plants is rarely reported. *Anemarrhena asphodeloides*, widely used in traditional medicine for thousands of years, exhibits a wide range of pharmacological activities including antitumor, anti-inflammatory, antiallergic, antibacterial, antiviral, antiaging effects (Liu et al., 2023; Lodi et al., 2025). Nevertheless, the use of fungal endophytes from *A. asphodeloides* in BMNP biosynthesis has not been invovled.

In this study, endophytic *Clonostachys rosea* ZMS36 isolated from the stems of medicinal plant *A. asophilus* was used to biosynthesize Ag-CuO NPs for the first time. The biologically synthesized Ag-CuO NPs were characterized and their antimicrobial and anticancer effects and biosafety were also evaluated.

## 2 Materials and methods

### 2.1 Isolation and identification of endophytic fungi

The endophytic fungus in this study was isolated from a healthy *A. asphodeloides* plant collected from the Taihang Mountains (34° 35′ N, 116° 27′ E) in China. The plant was identified by Professor Yizhu Chen at South China Botanical Garden, Chinese Academy of Sciences. Following the sterilization of the plant surface in accordance with the method proposed by Li et al. (2024), the fungal mycelium growing from the interior of the plant was collected and preserved on the slant of the PDA. Spore suspensions of endophytic fungi were maintained in a solution comprising 15% glycerol (v/v) at −80°C.

The endophytic fungal spores were revived on potato dextrose agar plates and placed in an incubator at 28°C for 1 week to allow for morphological observation and molecular analysis. The strain tissue was subsequently placed on a clean slide and the aerial mycelium and spores of the endophyte were subsequently observed via light microscopy (Kali et al., 2014). Molecular analysis was performed in accordance with the methodology described by Deng et al. (2014). DNA extracted from endophytes was amplified via the fungus-specific primers ITS1 (5′-TCCGTAGGTGAACCTGCGG-3′) and ITS4 (5′-TCCTCCGCTTATTGATATGC-3′). The qualified amplification products were subsequently sequenced at Sangon Biotech (Shanghai) Co., Ltd. The resulting sequences were subsequently matched in the NCBI GenBank database via the BLASTN program and neighbor-joining (NJ) analysis was conducted via MEGA software, with bootstrap values calculated from 1,000 repetitive runs.

### 2.2 Screening of endophytic fungi with the ability to synthesize Ag-CuO NPs

The endophytic fungal strains were cultivated in potato dextrose broth (PDB) for 96 h at 28°C with oscillation (120 rpm). The mycelium in the PDB was subsequently filtered through sterile filter paper and washed three times with sterilized deionized water (Zawadzka et al., 2021). Media-removed fungal mycelium (10 g) was resuspended in 100 mL of sterile deionized water for a period of 24 h at 28°C with agitation at a rate of 120 rpm. Ultimately, the culture filtrate was obtained through filtration via a syringe filter (BS-PES-22 Biosharp, China), which was employed in the synthesis of Ag-CuO NPs (Mistry et al., 2021).

The present study referenced the synthetic method developed by Sharma Deepika and colleagues for preliminary screening of endophytic fungi that are capable of synthesizing BMNPs. The first step involved mixing an equivalent amount of silver nitrate (AgNO_3_) and copper sulfate pentahydrate (CuSO_4_·5H_2_O) solution. The mixed solution of the two metal salts was added to the endophytic fungal culture filtrate under continuous magnetic stirring for BMNPs synthesis. The change in the color of the solution from colorless and transparent to black is indicative of the formation of bimetallic Ag–CuO NPs.

### 2.3 Biosynthesis and characterization of BMNPs

Optimization studies identified the optimal synthesis conditions by systematically varying metal salt concentrations (0.25 mM A^*g*+^ + 0.25 mM Cu^2+^ − 2.0 mM Ag^+^ + 2.0 mM Cu^2+^), reaction time (1–5 h), and pH (4–9). Under the optimized conditions, 10 mL of the two metal salts solution (0.5 mM Ag^+^ + 0.5 mM Cu^2+^) was added to 90 mL of fungal culture filtrate. The reaction mixture was adjusted to pH 6 and subjected to continuous magnetic agitation for 3 h at 85°C. The black BMNPs solutions were precipitated via centrifugation at 12,000 rpm for 30 min, after which they were washed three times with sterile deionized water. After centrifugation, the BMNPs were lyophilized for subsequent experiments.

The formation of NPs can be monitored via a UV-Vis spectrophotometer (Multiskan Skyhigh Thermo Fisher Scientific, China). The surface morphology of the Ag-CuO NPs was characterized by scanning electron microscopy (SEM; MIRA LMS TESCAN, Czech Republic) and their elemental composition was examined by energy dispersive spectroscopy (EDS). Transmission electron microscopy (TEM; JEM-F200 JEOL, Japan) allows further visualization of both the size and surface structure of the NPs. Fourier transform infrared spectroscopy (FTIR; Nicolet iN10 Thermo Fisher Scientific, USA) was used to investigate the surface functional groups of the Ag-CuO NPs synthesized from the fungal filtrates. The crystal structure of the Ag-CuO NPs was examined using X-ray diffraction (XRD; D8 Venture Bruker, Germany).

### 2.4 The antibacterial activity of Ag-CuO NPs

The antibacterial activity of the synthesized NPs was evaluated against seven human pathogenic bacteria. The following bacteria were included: *Pseudomonas aeruginosa* (ATCC27853)*, Staphylococcus aureus* (CMCC26003)*, Escherichia coli* (CMCC44102)*, Staphylococcus epidermidis* (ATCC49134)*, Salmonella typhimurium* (CMCC50115)*, Shigella dysenteriae* (CMCC51252), and methicillin-resistant *Staphylococcus aureus* (MRSA, ATCC43300). The MICs and MBCs of Ag-CuO NPs against each bacterial species were determined via broth microdilution in accordance with the Clinical Laboratory Standards Institute guidelines (CLSI, 2024). In summary, a stock solution of Ag-CuO NPs (or antibiotic solutions) was diluted to a concentration of 256 μg/mL and subsequently, two-fold serial dilutions were conducted until a concentration of 0.125 μg/mL was reached with MH broth. NPs solutions (or antibiotic solutions) of varying concentrations, with a volume of 100 μL per well, were added to 96-well microtiter plates. Ultimately, 100 μL of a pathogenic bacterial suspension (5 × 10^5^ CFU/mL) was inoculated into each well. Tigecycline and gentamicin were selected as positive controls for gram-positive and gram-negative bacteria, respectively. A further coadministration group was established for MRSA, in which Ag-CuO NPs and vancomycin antibiotics were administered concomitantly. Both agents were combined in equal volumes (v/v) at identical concentrations. The assay plates were incubated at 37°C for 16–20 h, after which the MICs were determined visually. Following the measurement of the MIC, suspensions from each well of the microtiter plate in which no observable bacterial growth was present were inoculated into MHA plates for the purpose of conducting the MBC test. The inoculated plates were incubated at 37°C for 20 h and the lowest concentration that did not result in colony colonies on MHA agar (i.e., which killed 100% of the bacterial population) was recorded as the MBC. The experiments were conducted in triplicate to ensure the reproducibility of the results.

### 2.5 The effects of Ag-CuO NPs on MRSA

Morphological changes of MRSA were observed by scanning electron microscopy. Sample preparation for SEM (JSM-IT800 JEOL, Japan) was carried out in accordance with the methodology proposed by Chew et al. (2018) with minor modifications. Freshly cultured bacterial suspensions of MRSA were adjusted to ~1 × 10^8^ CFU/mL. The bacteria were then treated with a 1 × MIC concentration of Ag-CuO NPs for 6 h at 37°C. A control group comprising bacteria that had not been treated with NPs was also established. The bacterial samples were subjected to centrifugation at 5,000 rpm for 3 min, after which they were washed three times with phosphate-buffered saline (PBS). The bacterial suspension was aspirated onto a slide and air dried. The slides were then fixed with 2.5% glutaraldehyde for 24 h. The fixed samples were dehydrated in graded ethanol (30%, 50%, 80%, 90%, 100%) for 10 min each time. The slides were left to dry overnight and then sputter-coated in preparation for observation via SEM.

The ability of Ag-CuO NPs to prevent or reduce MRSA biofilm formation was assessed according to the following methodology. The pathogenic bacterial strains were cultivated in LB broth containing 0.5% glucose for 18 h at 37°C. Thereafter, the bacterial suspension (10^8^ CFU/mL) was added to polystyrene 96-well plates at a volume of 200 μL per well and 1/2, 1/4, or 1/8 MIC (final concentration) of Ag-CuO NPs was dispensed into the different wells (Ibrahim et al., 2021). The bacterial biofilms were grown at 37°C for 48 h, after which the medium was removed. To remove floating bacteria, the 96-well plates were washed three times with phosphate-buffered saline (PBS) at a pH of 7.4. The formed biofilm was then fixed with 200 μL of 95% methanol for 10 min in each well and 0.3% crystal violet was added to the wells for staining. Thereafter, the plates were rinsed with distilled water and air dried (Kot et al., 2018). For the quantitative biofilm formation assays, 30% acetic acid was added to the wells and the absorbance was measured at OD_570nm_ via a microplate reader. The biofilm inhibition rate was calculated according to the following formula:


$$\begin{array}{l} \text{Biofilm inhibition }(\%)=\\ \;\frac{\text{Control }{\text{OD}}_{\text{570nm}}\;-\text{ Treated }{\text{OD}}_{\text{570nm}}}{\text{Control }{\text{OD}}_{\text{570nm}}}\times 100\% \end{array}$$

The expression of the antimicrobial resistance gene (*mecA*) was identified through the use of quantitative real-time polymerase chain reaction (PCR). MRSA was treated with 1/2 MIC concentrations of Ag-CuO NPs, vancomycin or Ag-CuO NPs + vancomycin (1:1 = v:v) for 6 h at 37°C. The untreated sample served as a negative control. Total bacterial RNA was extracted via the RNA Extraction Kit (Easun Biotech, China) and quantified via the A260 method via a NanoDrop Lite spectrophotometer (Multiskan Skyhigh, Thermo Fisher Scientific, China). The RNA was then transcribed into cDNA via a reverse transcription kit (HiScript III All-in-one RT SuperMix Perfect for qPCR Vazyme, China). Real-time PCR experiments were conducted in accordance with the instructions provided with the fluorescence quantification kit (TB Green^®^ Premix EX Taq™ II, Tli RNaseH Plus Takara Biomedical Technology, China). In this study, the *RpoB* gene, which encodes an RNA polymerase subunit, was used as the internal reference and specific primers were designed (F-CAGCTGACGAAGAAGATAGCTATGT, R-ACTTCATCATCCATGAA ACGACCAT; Kot et al., 2018). The specific primers used for the *mecA* gene were previously described (F-ATCCACCCTCAAACAGGTGAAT, R-GGAACTTGTTGAGCAGAG GTTC; Wang and Nicholaou, 2017). The following thermal cycling conditions were used for RT–PCR amplification: initial denaturation at 95°C for 30 s, followed by 40 cycles of annealing at 95°C for 5 s and extension at 60°C for 30 s. The relative expression of the target genes can be calculated via the following formula: RQ = 2^−Δ*ΔCt*^ (Abdelraheem et al., 2021).

### 2.6 The anticancer activity of Ag-CuO NPs

#### 2.6.1 Cell culture

The following cancer cell lines were utilized in this study: A549 (a human lung cancer cell line), PDSF (a pituitary tumor-derived folliculostellate cell line) and HeLa (a human cervical cancer cell line). The cancer cells were maintained in culture in DMEM containing 10% fetal bovine serum and 1% penicillin–streptomycin at 37°C and 5% CO_2_.

#### 2.6.2 Viability of cells

The toxicity of Ag-CuO NPs toward cancerous cells was assessed by determining their effect on cell viability through the use of a Cell Counting Kit-8 (CCK-8; Solarbio, China) assay. Different cancer cell lines were inoculated into 96-well plates at a density of 0.8 × 10^4^ cells/well and incubated at 37°C for 24 h. Next, the cells were treated with various concentrations of Ag-CuO NPs (0.25, 0.5, 1, 2, 4, 8, 16, 32, 64, and 128 μg/mL) for a period of 24 h, with cells not exposed to NPs serving as negative controls. To each well, 10 μL of the CCK-8 solution was added and the mixture was incubated for 1 h at 37°C. Absorbance measurements at OD_450nm_ were conducted via a microplate reader, with a blank background group (containing only DMEM) established (Tian et al., 2020). The relative cell proliferative activity was calculated as follows: Viability of cells (%) = (Treated OD_450nm_ − Blank OD_450nm_)/(Control OD_450nm_ − Blank OD_450nm_) × 100%.

The half-maximal inhibitory concentration (IC_50_) was determined as the concentration of Ag-CuO NPs required to inhibit 50% of cell growth. The IC_50_ values were calculated through linear regression analysis performed using GraphPad Prism8 software (Pesic et al., 2015).

#### 2.6.3 *In vitro* scratch test

The objective of the scratch test was to detect the effects of Ag-CuO NPs on cell–cell interactions and cell migration. The HeLa cells with the lowest IC_50_ values, as determined by the cell proliferation assay, were selected for testing the impact of Ag-CuO NPs on their migratory capacity. HeLa cells were inoculated into 12-well plates at a density of 1 × 10^7^ cells/well and incubated at 37°C in a 5% CO_2_ incubator for 24 h to allow growth into a confluent monolayer. A 200 μL sterile pipette tip was subsequently used to create a scratch in the cell mixture. The cells in the 12-well plates were treated with Ag-CuO NPs at concentrations that had an IC_50_ value for increasing HeLa cell viability. Additionally, wells without NPs were used as controls. The samples were observed and photographed under an inverted microscope (Soptop ICX41, China) at 12 h intervals over a period of 48 h. The dimensions of the gap were quantified through analysis with ImageJ software (Kumari et al., 2021).

#### 2.6.4 Angiogenesis inhibition capacity detected by Hen's egg test-chorioallantoic membrane (HET-CAM) assay

The ability of Ag-CuO NPs to inhibit angiogenesis in chicken embryos was investigated via the method proposed by Mani et al. (2021). A small hole was opened in the air sac of a 9-day-old chick embryo (Zhaoqing Dahuanong Biology Medicine Co., Ltd., China) via forceps. Different concentrations of Ag-CuO NPs solution (100, 200, and 300 μg/mL) were then added to the surface of the CAM. Prior to the placement of the samples, the number of blood vessels in each egg was quantified. The process of angiogenesis within the eggs was subsequently observed and evaluated after 2 and 18 h. The positive control was 0.1 N NaOH, while 0.9% NaCl was used as the negative control (Mani et al., 2021).

### 2.7 Cytotoxicity studies

#### 2.7.1 *In vitro* cytotoxicity assay

Human epidermal keratinocyte HaCaT cells were cultured in complete medium (DMEM containing 10% FBS and 1% penicillin–streptomycin) at 37°C with 2.5% CO_2_. The cells were inoculated into 96-well microtiter plates at a density of 0.8 × 10^4^ cells/well and incubated for 24 h to enable adhesion to the wall. Then, different concentrations of Ag-CuO NPs (0.25, 0.5, 1, 2, 4, 8, 16, 32, 64, and 128 μg/mL) were added and a control group without NPs was set up (Shkryl et al., 2021). CCK-8 analyses and cell viability measurements and calculations were also conducted in accordance with previously described methods (Tian et al., 2020).

#### 2.7.2 Hemolysis assay

A study of the cytotoxic effects of Ag-CuO NPs used a hemolysis assay. Sheep erythrocytes (Guangzhou Hongquan Biotechnology Co., Ltd., China) were treated with different concentrations of NPs for 1 h at 37°C. The concentrations were selected to represent the MIC, 1/2 MIC, and 1/4 MIC of MRSA. The sheep erythrocytes were treated with Triton X-100 (0.1%) as a positive control and with PBS as a negative control. Following the incubation period, the samples were subjected to centrifugation for 10 min, after which the absorbance of the supernatant was measured at a wavelength of 450 nm (Kamli et al., 2021). The rate of erythrocyte hemolysis was calculated via the following formula:


$$\text{Hemolysis rate }(\%)\;=\;\frac{\begin{array}{c} \text{Treated }{\text{OD}}_{\text{450nm}}\;-\\ \text{Negative control }{\text{OD}}_{\text{450nm}} \end{array}}{\begin{array}{c} \text{Positive control }{\text{OD}}_{\text{450nm}}\;-\\ \text{Negative control }{\text{OD}}_{\text{450nm}} \end{array}}\times 100\%$$

### 2.8 Statistical analysis

All experiments were independently repeated three times under identical conditions. Data were expressed as mean ± standard deviations (SD). Statistical analyses were performed with GraphPad Prism 8.0 software and the resulting data were compared via one-way analysis of variance (ANOVA). A *p* value < 0.05 was considered statistically significant.

## 3 Results

### 3.1 Isolation and identification of endophytic fungi with the ability to biosynthesize BMNPs

A total of 31 strains of endophytic fungi were isolated from *A. asphodeloides*. Among them, the aqueous extract of the mycelium of ZMS36 caused a color change in response to the addition of a solution of AgNO_3_ and CuSO_4_·5H_2_O, resulting in a shift from colorless to black. These results indicated that the endophytic strain ZMS36 likely has the capacity to biosynthesize bimetallic NPs.

**Figure 1:**
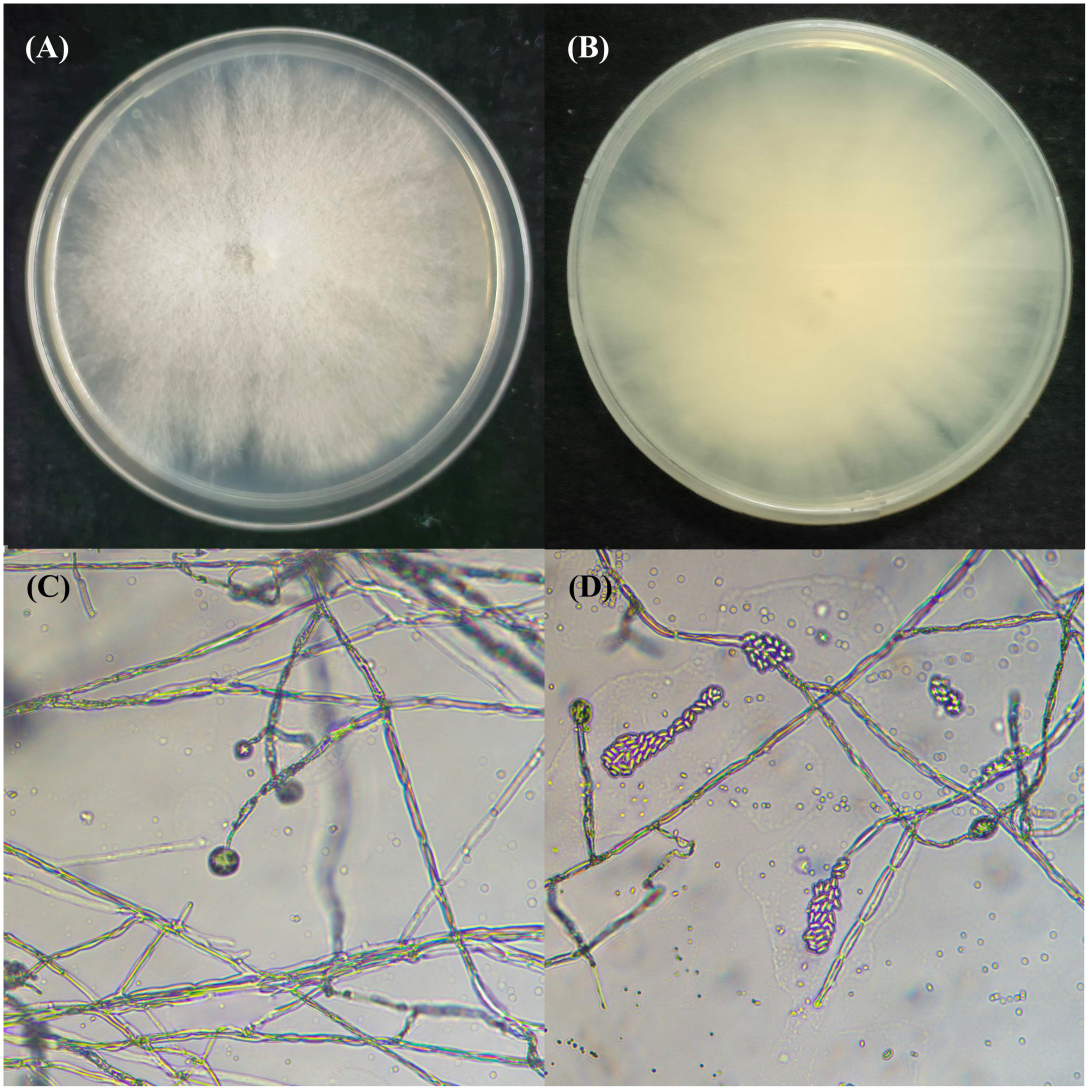
Morphology of endophytic *C. rosea* ZMS36. **(A)** The front of the fungal colony. **(B)** The back of the fungal colony. **(C)** The microscopic conidiophores of *C. rosea* ZMS36. **(D)** The conidia of *C. rosea* ZMS36.

**Figure 2:**
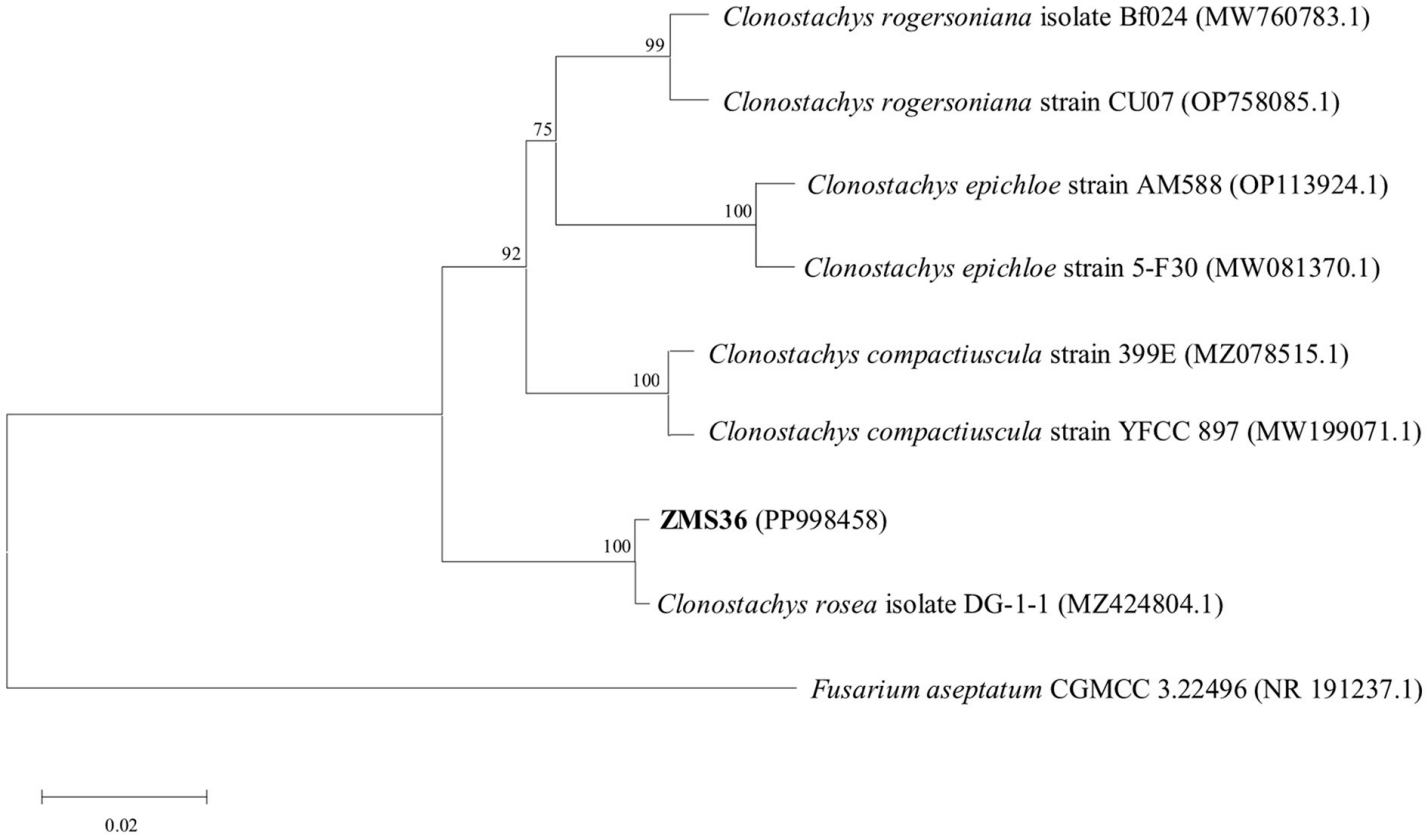
The phylogenetic tree based on the ITS rDNA gene sequence of *C. rosea* ZMS36. The phylogenetic tree was constructed by Neighbor-Joining method. *Fusarium aseptatum* CGMCC 3.22496 (NR 191237.1) was used as the out-group. The numbers on the phylogenetic tree were the bootstrap values after 1,000 replicates and the scale length represented the genetic distance.

The endophytic fungus ZMS36 had a slow growth rate. The colony presented a white, fluffy appearance on the front side and produced yellow pigmentation on the back side (Figures 1A, B). Strain ZMS27 had septal mycelia, conidiophores with broom-like branches and colorless oval conidia (Figures 1C, D). BLAST analysis revealed that the ITS gene sequence of strain ZMS36 (GenBank accession number: PP998458) shared 99.81% similarity with that of *C. rosea* (MZ424804.1), which was isolated from roots of medicinal plant *Angelica sinensis* in the village of Sada, Minhe County, Haidong City, in Qinghai Province, China (36° 19′ 9.47″ N, 102° 51′ 30.83″ E, elevation: 2,437 m; Ma et al., 2022). The phylogenetic tree indicated that strain ZMS36 clustered well together with 7 representative taxa of *Clonostachys*. Within this clade, strains ZMS36 and *C. rosea* (MZ424804.1) formed a subclade with relatively strong bootstrap support of 100% (Figure 2). On the basis of morphological characteristics and phylogenetic analysis, strain ZMS36 was categorized as *C. rosea*.

### 3.2 Biosynthesis and characterization of Ag-CuO NPs

As shown in Supplementary Figure S1A, Ag-CuO NPs could be successfully synthesized by strain ZMS36 at 0.5 mM Ag^+^ + 0.5 mM Cu^2+^ when the pH was 6 and the reaction time was 3 h. However, no NPs were synthesized at salt concentrations of 0.25 mM Ag^+^ + 0.25 mM Cu^2+^, 1.0 mM Ag^+^ + 1.0 mM Cu^2+^, and 2.0 mM Ag^+^ + 2.0 mM Cu^2+^. The Ag-CuO NPs were synthesized at reaction time of 3, 4, and 5 h under pH 6 and salt concentrations of 0.5 mM Ag^+^ + 0.5 mM Cu^2+^, with average particle sizes of 36.86, 49.67, and 58.26 nm, respectively (Supplementary Figure S1B). In contrast, no NPs were synthesized at reaction times of 1 or 2 h. The results showed that the optimal reaction time for the synthesis of Ag-CuO NPs by strain ZMS36 was 3 h. Furthermore, Ag-CuO NPs could only be biosynthesized at pH 6 and pH 7, with average particle sizes of 36.99 and 57.34 nm, respectively (Supplementary Figure S1C). Therefore, the optimized reaction conditions for the synthesis of Ag-CuO NPs by strain ZMS36 were pH 6, a reaction time of 3 h, and salt concentrations of 0.5 mM Ag^+^ + 0.5 mM Cu^2+^. The Ag-CuO NPs biosynthesized at the optimized reaction conditions were used for the further study.

**Figure 3:**
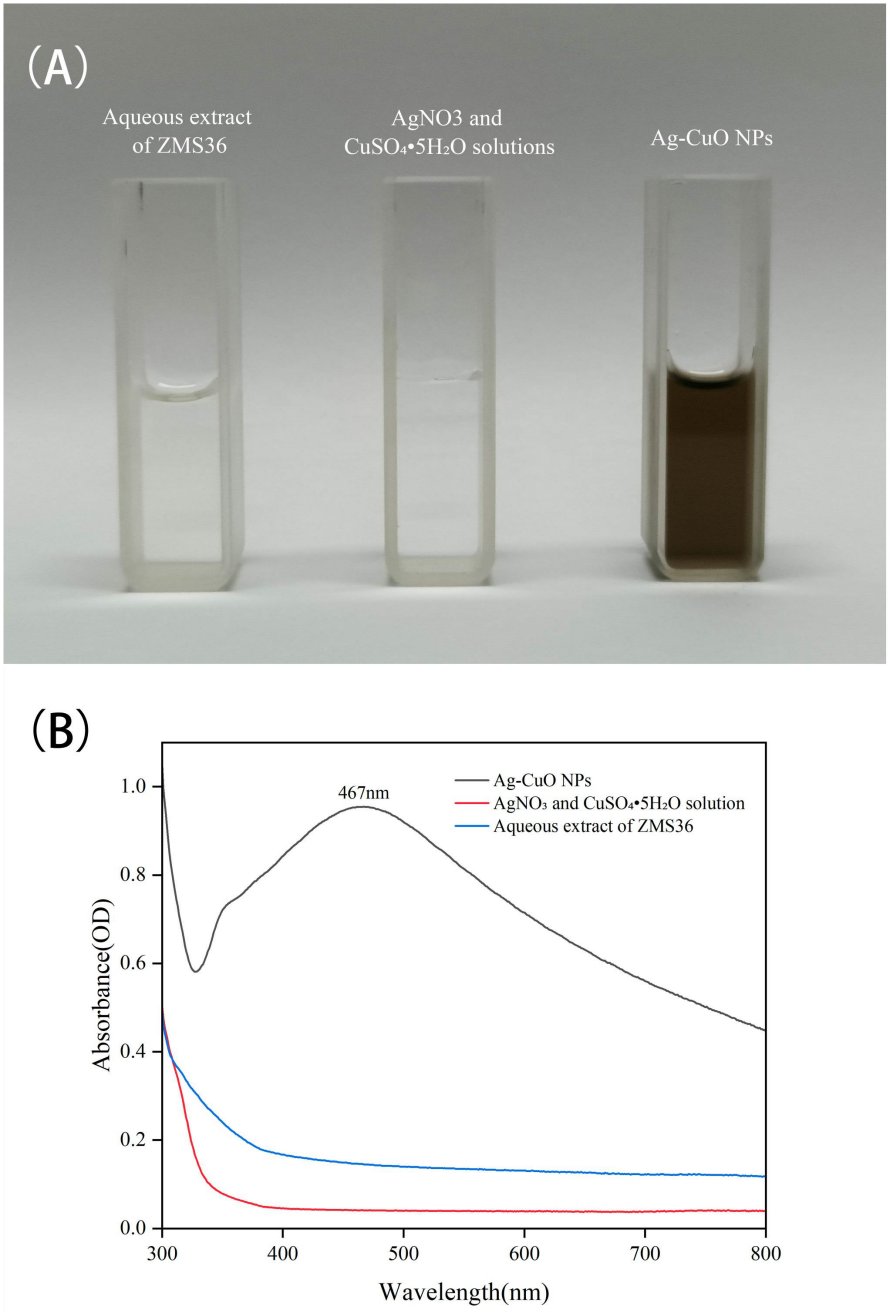
The synthesis of Ag-CuO NPs using the aqueous extract of endophytic *C. rosea* ZMS36. **(A)** The color change of solutions in the synthesis of Ag-CuO NPs. **(B)** UV–Visible spectrum of Ag-CuO NPs.

**Figure 4:**
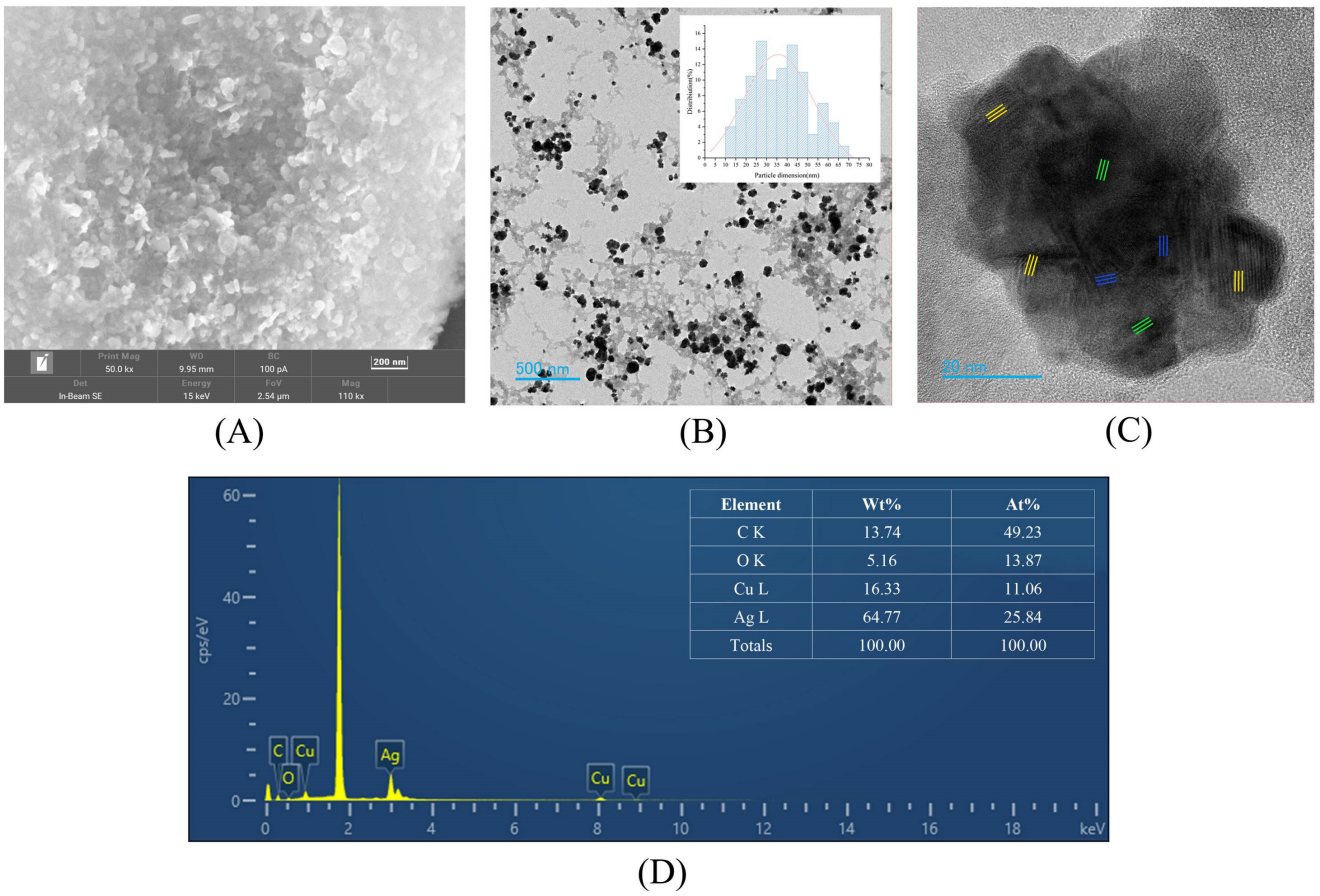
Characterization of Ag-CuO NPs. **(A)** SEM image of Ag-CuO NPs. **(B)** TEM image of Ag-CuO NPs and their size distribution. **(C)** High-resolution TEM micrographs of Ag-CuO NPs. The yellow and blue parts are related to the Ag lattice region and the Cu lattice region, respectively, and the green part is related to the Ag+Cu alloy region. **(D)** EDS analysis of Ag-CuO NPs.

**Figure 5:**
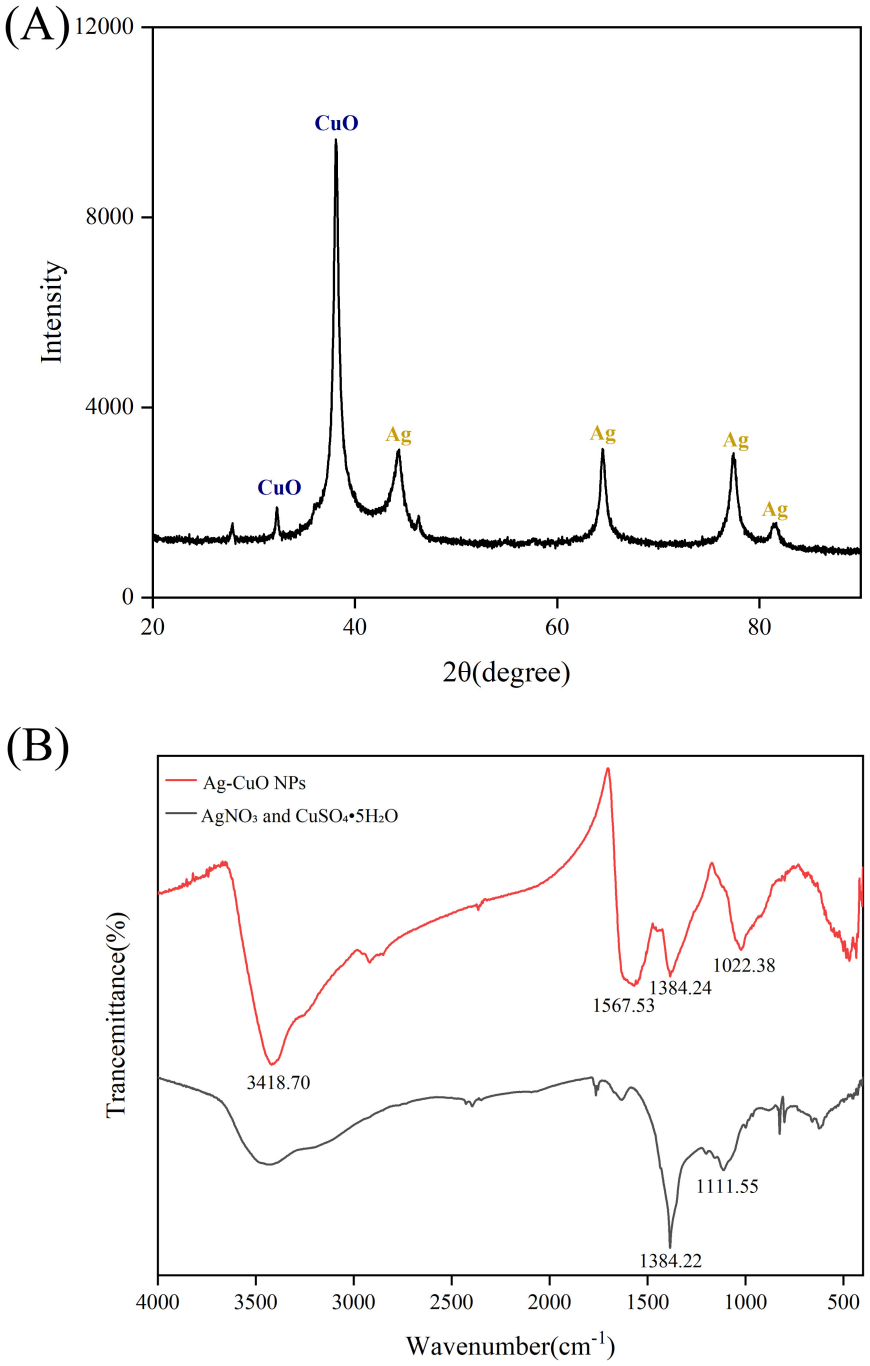
The XRD pattern **(A)** and FTIR spectrum **(B)** of Ag-CuO NPs.

During the synthesis process, the color of the solution changed from colorless to black (Figure 3A). The UV-vis spectrum indicated that the reaction mixture exhibited a pronounced absorption peak at ~467 nm (Figure 3B). These results indicated that Ag-CuO NPs were successfully synthesized by strain ZMS36. The SEM and TEM results indicated that the NPs exhibited an irregularly spherical morphology with slight aggregation (Figures 4A, B). The size distribution of the NPs ranged from 11.11 to 69.10 nm, with an average size of 36.63 nm (Figure 4B). The biosynthesized NPs had regions composed of silver, copper, or a combination of the two metals on their surfaces (Figure 4C). The results of EDS analysis confirmed the presence of Ag, Cu, C, and O in the biosynthetic NPs (Figure 4D). XRD of the Ag-CuO NPs revealed six diffraction peaks at 32.30°, 38.07°, 44.39°, 64.51°, 77.37°, and 81.32° (Figure 5A). The XRD pattern of Ag-CuO NPs exhibited six distinct diffraction peaks at 32.30°, 38.07°, 44.39°, 64.51°, 77.37°, and 81.32° (Figure 5A). Among these, the peaks at 44.39°, 64.51°, 77.37°, and 81.32° were identified as corresponding to the crystallographic planes (200), (220), (311), and (222), respectively, confirming the presence of metallic silver within the Ag-CuO NPs structure (Al-Haddad et al., 2019). Additionally, the presence of CuO was confirmed by the diffraction peaks at 32.30° and 38.07°, which corresponded to the crystallographic planes (110) and (111), respectively (Rosbero and Camacho, 2017). As confirmed by the XRD analysis, the biosynthesized Ag-CuO NPs exhibited a crystalline nature, characterized by a face-centered cubic (fcc) structure combined with a monoclinic phase (Al-Haddad et al., 2019; Rosbero and Camacho, 2017).

The FTIR analysis revealed distinct differences in the spectra of the metal salt mixture (AgNO_3_ and CuSO_4_·5H_2_O) and the Ag-CuO NPs (Figure 5B). The metal salt mixture exhibited characteristic absorption peaks at 1384.22 cm^−1^ (${\text{NO}}_{3}^{-}$ stretching vibration) and 1111.55 cm^−1^ (${\text{SO}}_{4}^{2-}$ symmetric stretching), while the Ag-CuO NPs spectrum displayed distinct absorption peaks at 3418.70, 1567.53, 1384.24, and 1384.24 cm^−1^. The absorption band at 3418.70 cm^−1^ was attributed to the stretching vibrations of -OH and -NH groups (Manikandan et al., 2023). The peak at 1567.53 cm^−1^, which was primarily attributed to the bending vibrations of aromatic C=C bonds (Manikandan et al., 2023). The absorption band at 1384.24 cm^−1^ was associated with the bending vibrations of the aldehyde functional group (–CH_3_; Abdillah et al., 2017). The peak at 1022.38 cm^−1^ was indicative of the symmetric C-O-C stretching vibrations (Sengupta et al., 2015). The results indicated that the formation of Ag-CuO NPs was associated with various secondary metabolites (such as proteins, polysaccharides, and organic acids) secreted by the endophytic fungus *C. rosea*.

### 3.3 The antibacterial effects of the Ag-CuO NPs

**Figure 6:**
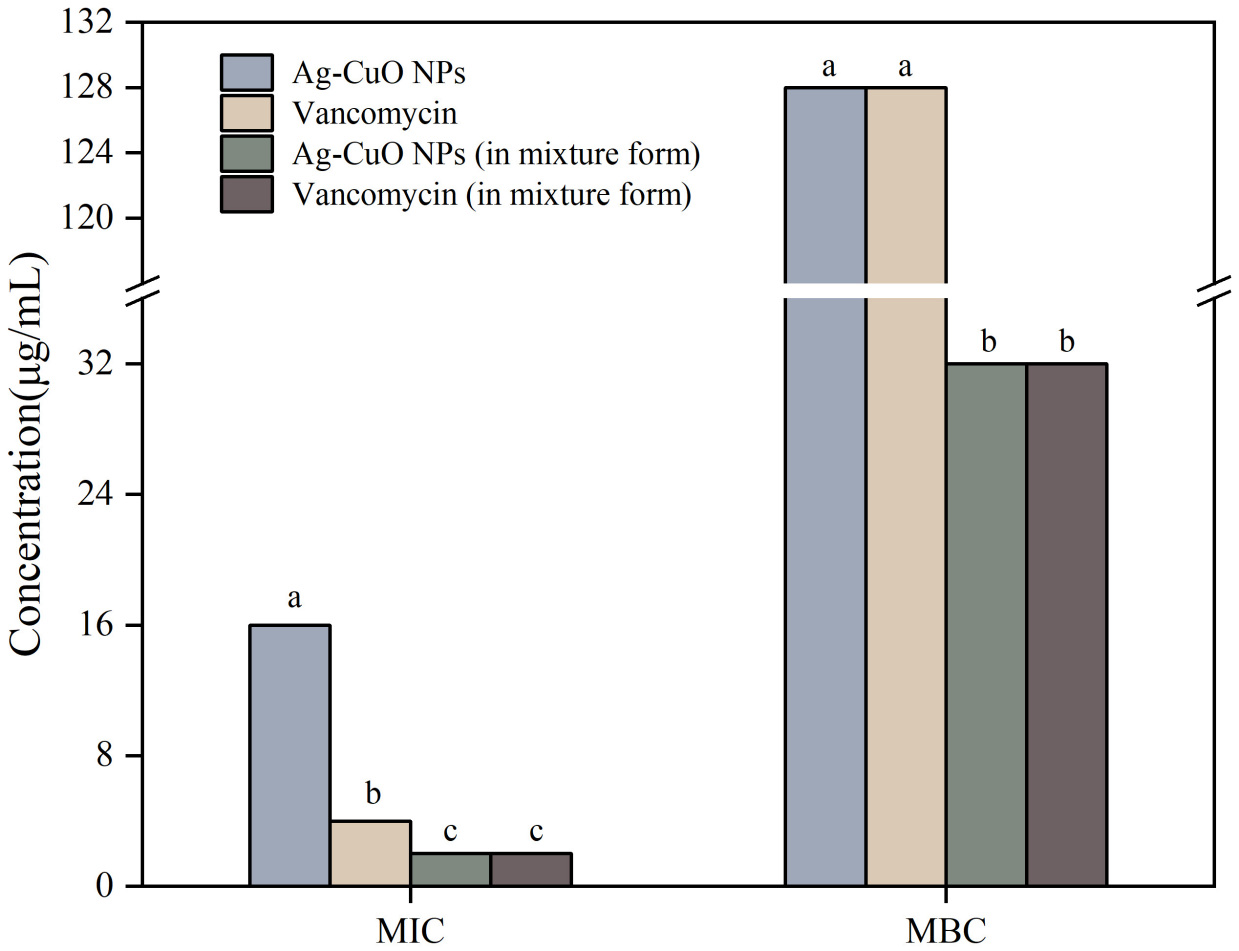
The MICs and MBCs of Ag-CuO NPs against MRSA. The vertical line on each bar shows the standard deviation (n = 3). Different lowercase letters indicated significant difference in MIC/MBC values among different drugs against the same bacteria (*p* < 0.05).

The Ag-CuO NPs exhibited good antibacterial activity (Table 1). The MICs of Ag-CuO NPs against *P. aeruginosa, S. aureus, E. coli, S. typhimurium, S. dysenteriae*, and *S. epidermidis* were 8, 8, 4, 4, 4, and 1 μg/mL, respectively, while the MBCs were 64, 64, 16, 16, 32, and 16 μg/mL, respectively (Table 1). The Ag-CuO NPs also showed good inhibitory activity against MRSA. The MICs and MBCs of Ag-CuO NPs against MRSA were 16 and 128 μg/mL, respectively. The MIC and MBC of Ag-CuO NPs in conjunction with vancomycin were reduced by 87.50% and 75.00%, respectively, compared with those of Ag-CuO NPs alone (Figure 6). Although, the antibacterial capacity of the Ag-CuO NPs was lower than that of the positive controls that were the antibiotics currently in clinical use, they exhibited good broad-spectrum antibacterial activity, particularly showing strong resistance against MRSR.

**Table 1:** The MICs and MBCs of Ag-CuO NPs against pathogenic bacteria.

**Bacteria**	**Drug**	**MIC (μg/mL)**	**MBC (μg/mL)**
*P. aeruginosa*	Ag-CuO NPs	8^a^	64^a^
	Gentamicin	1^b^	2^b^
*S. aureus*	Ag-CuO NPs	8^a^	64^a^
	Tigecycline	0.125^b^	4^b^
*E. coli*	Ag-CuO NPs	4^a^	16^a^
	Gentamicin	0.125^b^	0.5^b^
*S. paratyphi*	Ag-CuO NPs	4^a^	16^a^
	Gentamicin	0.25^b^	0.5^b^
*S. dysenteriae*	Ag-CuO NPs	4^a^	32^a^
	Gentamicin	0.25^b^	0.5^b^
*S. epidermidis*	Ag-CuO NPs	1^a^	16^a^
	Tigecycline	0.25^b^	1^b^

Different lowercase letters indicated significant difference in MIC/MBC values among different drugs against the same bacteria (*p* < 0.05).

**Figure 7:**
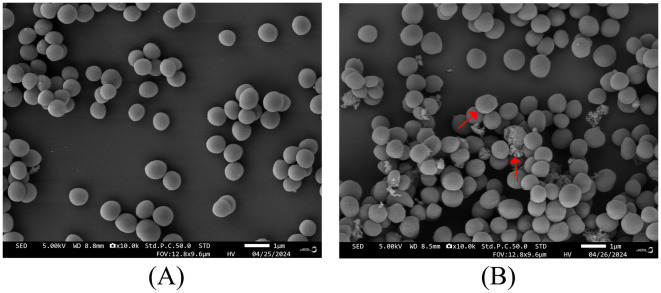
SEM images of MRSA treated with Ag-CuO NPs. **(A)** The untreated control. **(B)** The treatment with Ag-CuO NPs.

**Figure 8:**
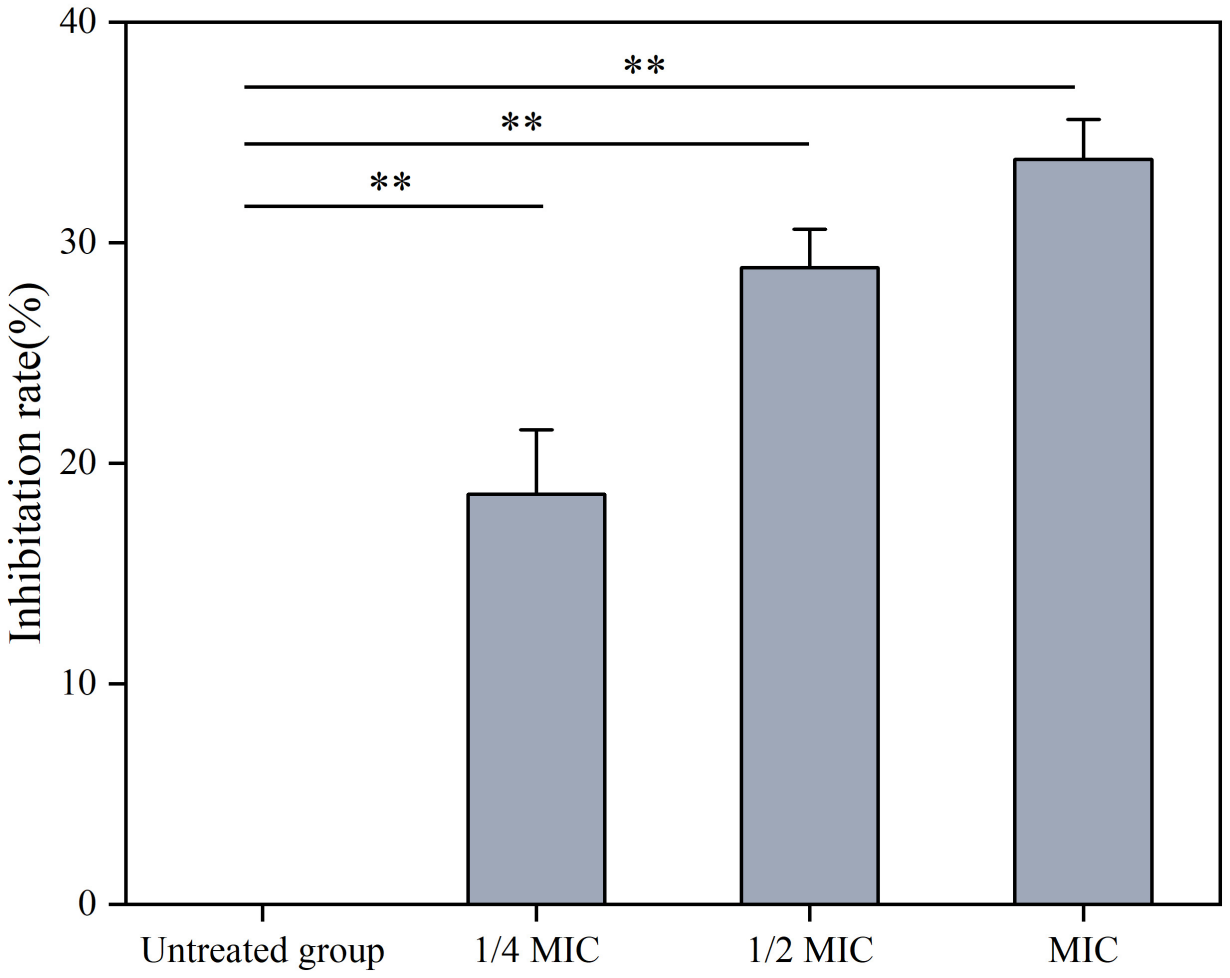
The effects of Ag-CuO NPs on the synthesis of MRSA biofilm. The vertical line on each bar shows the standard deviation (n = 3). **p < 0.01.

**Figure 9:**
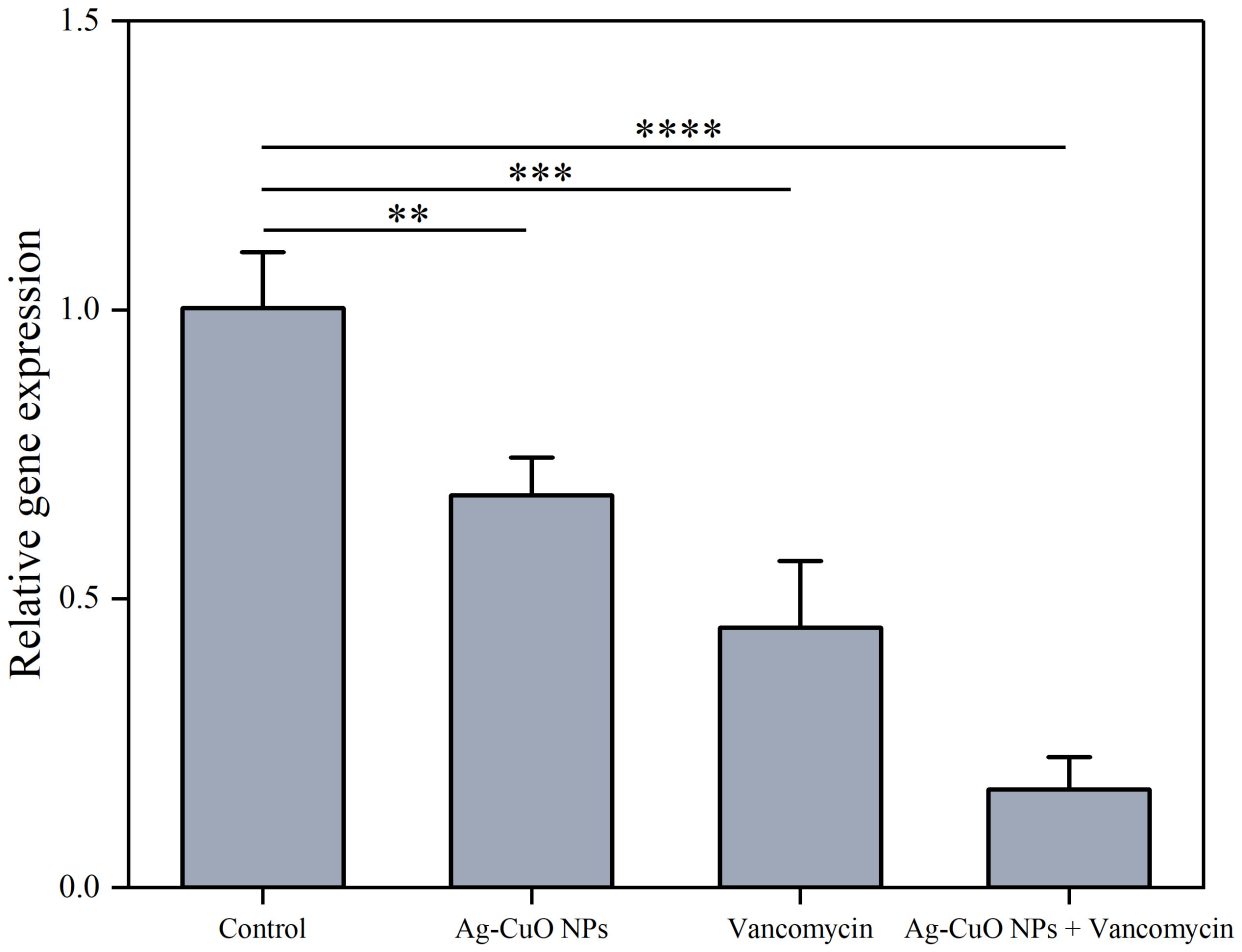
The effects of Ag-CuO NPs on the expression of the MRSA resistance gene *mecA*. The vertical line on each bar shows the standard deviation (n = 3). **p < 0.01, ***p < 0.001, and ****p < 0.0001.

The SEM results indicated that the treatment with Ag-CuO NPs caused notable changes in the cellular structure of MRSA, including rupture and collapse of the cell membrane, as well as cellular content leakage (Figure 7). The Ag-CuO NPs suppressed the biofilm formation of MRSA by 33.78% at the MIC treatment and 18.61% at 1/4 of the MIC treatment (Figure 8). The expression level of the MRSA resistance gene *mecA* was significantly lower in the Ag-CuO NP treatment group compared to the control group (*p* < 0.01). Furthermore, the combination treatment of vancomycin and Ag-CuO NPs led to a 5.92-fold downregulation of *mecA* gene expression compared to treatment with Ag-CuO NPs alone (*p* < 0.0001; Figure 9).

### 3.4 The antitumor and the anti-angiogenic activities of the Ag-CuO NPs

**Figure 10:**
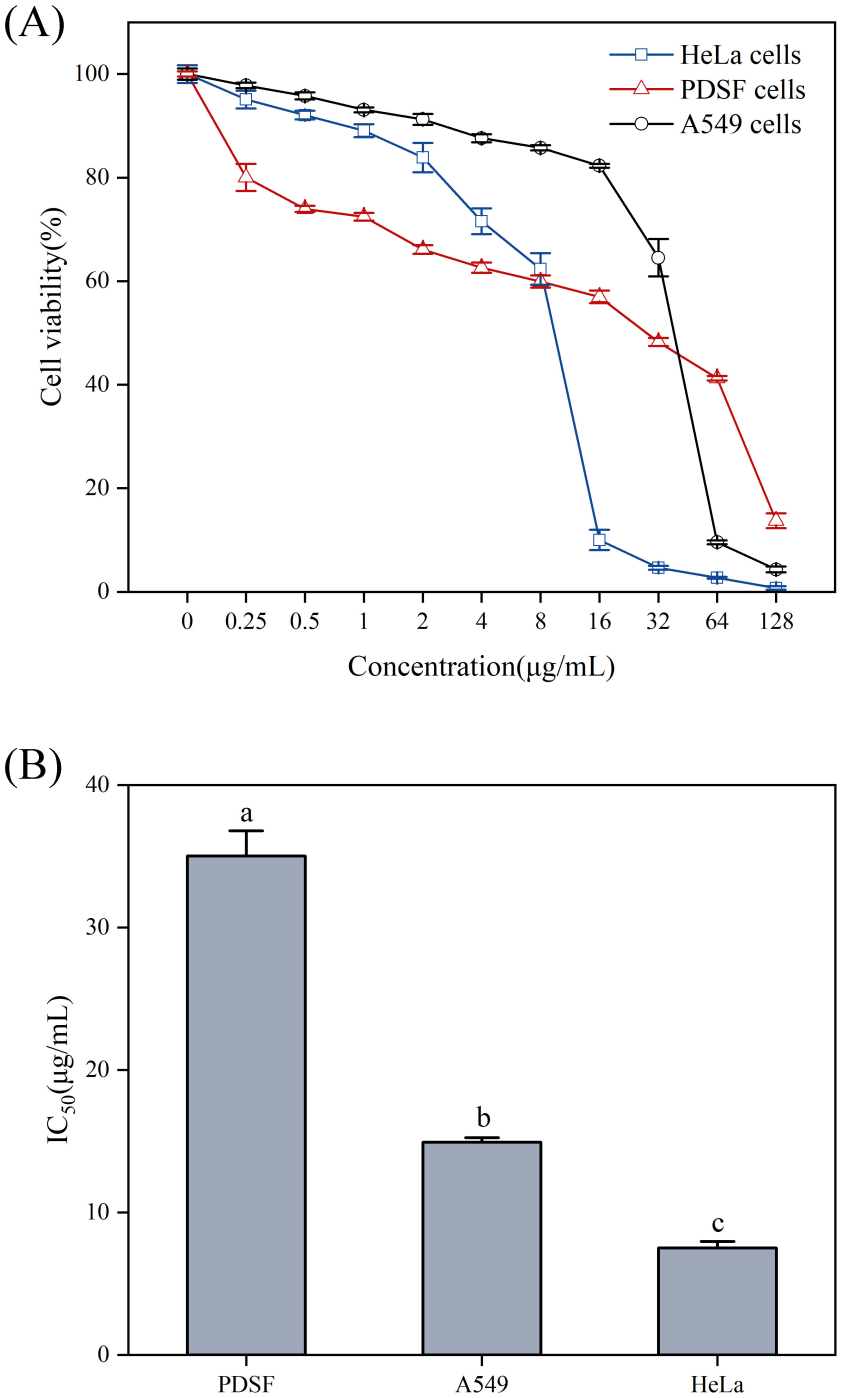
The antiproliferative activity **(A)** and IC_50_ values **(B)** of Ag-CuO NPs against tumor cell lines. The vertical line on each bar shows the standard deviation (n = 3). Different lowercase letters indicate statistically significant differences in IC_50_ values (*p* < 0.05) among cancer cell lines.

**Figure 11:**
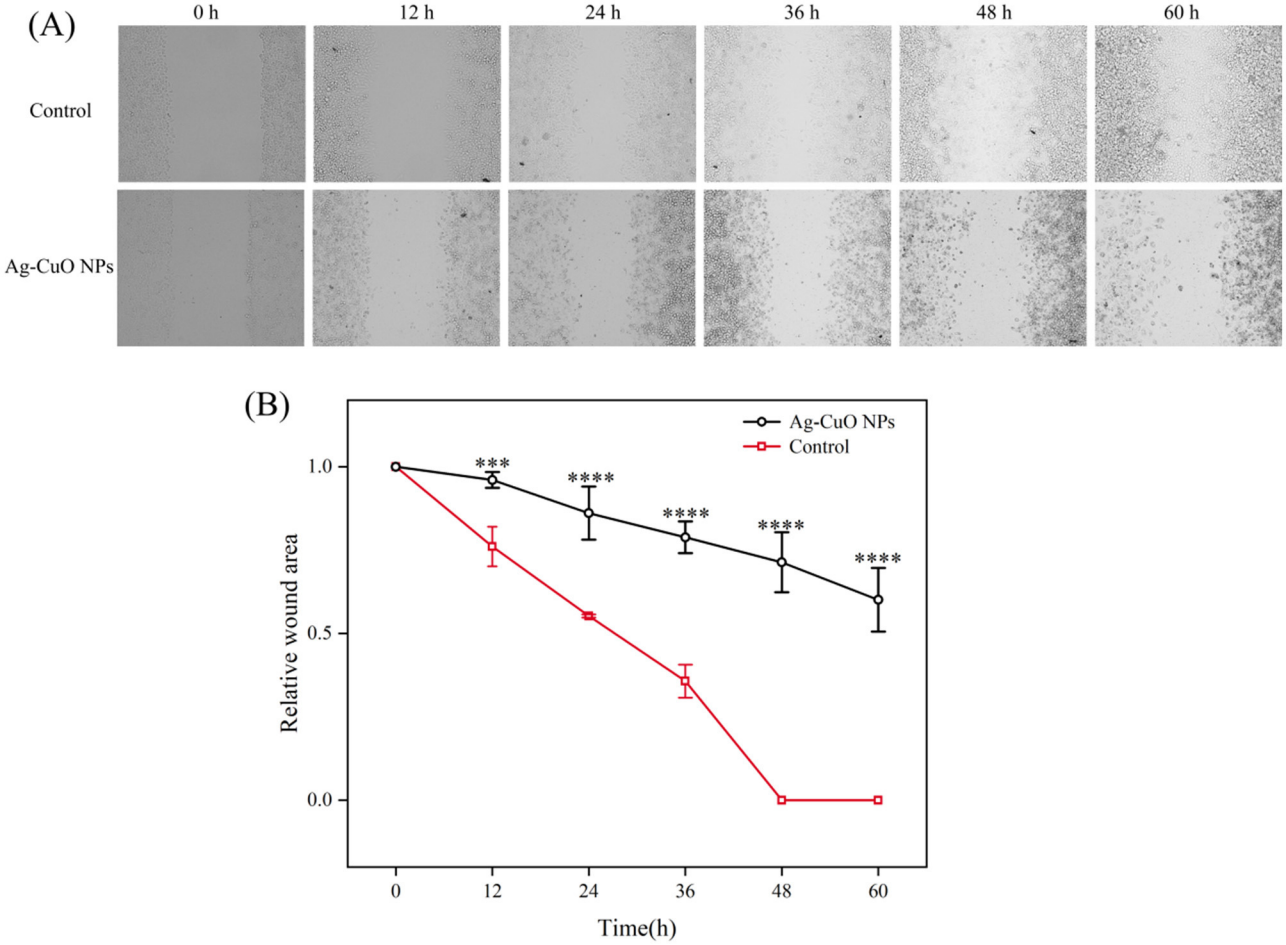
The inhibitory effect of Ag-CuO NPs on the migration of HeLa cells. **(A)** Microscopy images of the cell scratch test. **(B)** Relative scratch area in the cell scratch test after treatment of HeLa cells with Ag-CuO NPs. The vertical line on each bar shows the standard deviation (n = 3). ***p < 0.001 and ****p < 0.0001.

**Figure 12:**
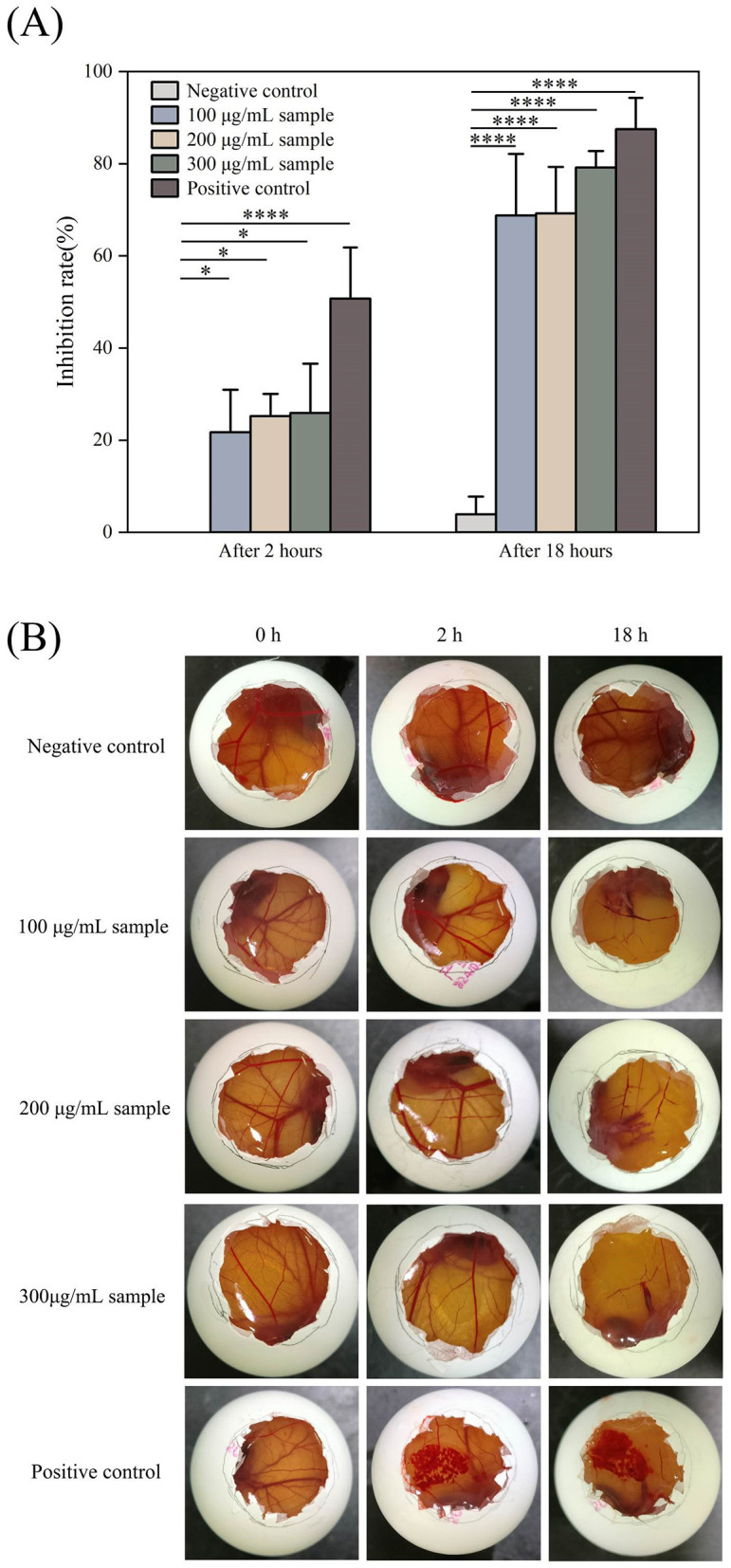
The Anti-angiogenic activity of Ag-CuO NPs. **(A)** The anti-angiogenic rates of different concentrations of Ag-CuO NPs after 2 and 18 h treatment. PBS and NaOH were used as the negative and positive controls, respectively. The vertical line on each bar shows the standard deviation (n = 3). *p < 0.05 and ****p < 0.0001. **(B)** The photographs of HET-CAM after the addition of different samples.

As shown in Figure 10A, the Ag-CuO NPs exhibited pronounced antiproliferative activity against three tumor cells. The IC_50_ values for the HeLa, PDSF, and A549 cells were 7.53, 35.03, and 14.93 μg/mL, respectively (Figure 10B). Among them, Ag-CuO NPs showed the most pronounced antiproliferative activity against HeLa cells. The *in vitro* scratch assay revealed that, following a 48-h treatment with Ag-CuO NPs, the scratch area was 71.36% larger than that of the control group (*p* < 0.0001), indicating their potent capacity to inhibit the migration of HeLa cell (Figure 11). The inhibition rates of angiogenesis after 2 h of treatment with Ag-CuO NPs at concentrations of 100, 200, and 300 mg/mL were 21.74%, 25.24%, and 25.91% (*p* < 0.05), respectively. After 18 h of treatment, the inhibition rates increased to 68.81%, 69.26%, and 79.18% (*p* < 0.0001), respectively (Figure 12).

### 3.5 Biosafety analysis of Ag-CuO NPs

**Figure 13:**
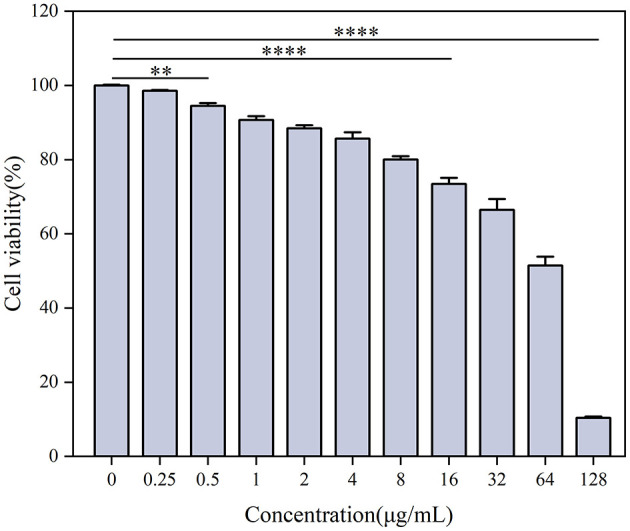
The cytotoxic effects of Ag-CuO NPs on HaCat cells. The vertical line on each bar shows the standard deviation (n = 3). **p < 0.01 and ****p < 0.0001.

**Figure 14:**
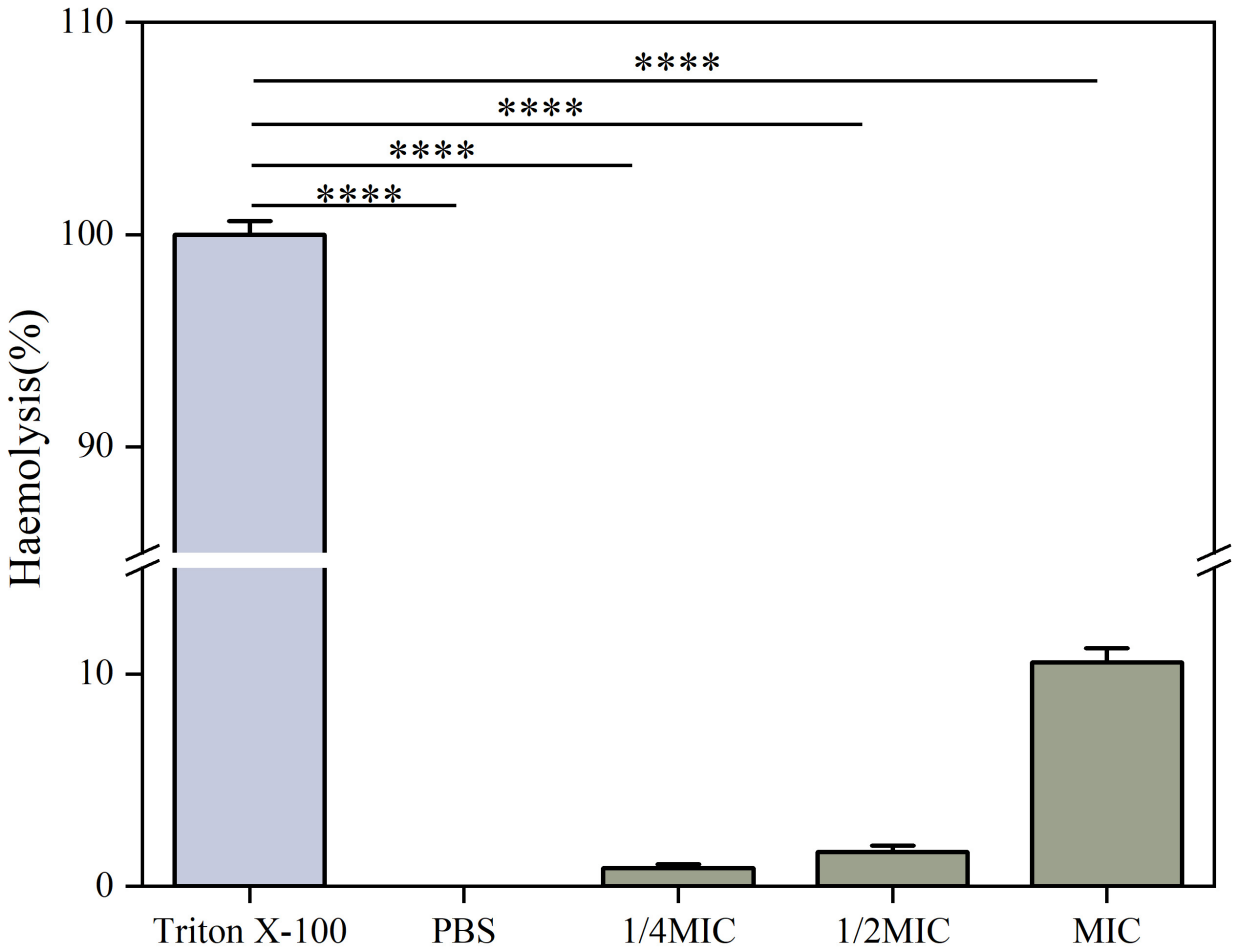
The hemolytic activity of Ag-CuO NPs. PBS was used as the negative control and Triton X-100 as the positive control. The vertical line on each bar shows the standard deviation (n = 3). ****p < 0.0001.

The cytotoxicity of Ag-CuO NPs to the HaCat cells was determined using the CCK-8 assay. The viability of the HaCat cells decreased with increasing concentrations of NPs. There was no significant cytotoxicity to the HaCat cells at concentrations not exceeding 16 μg/mL (Figure 13). The effect of Ag-CuO NPs on the hemolysis of sheep erythrocytes was also evaluated. The results showed the hemolysis rates of Ag-CuO NPs at 1/4 MIC (4 μg/mL), 1/2 MIC (8 μg/mL), and MIC (16 μg/mL) values against MRSA were 0.85%, 1.61% and 10.54% (*p* < 0.0001), respectively. The results indicated that the Ag-CuO NPs within the MIC ranges demonstrated a markedly low degree of cytotoxicity compared with the positive control (100% hemolysis rate; Figure 14).

## 4 Discussion

To date, the synthesis of BMNPs has primarily relied on chemical methods, with limited applications of plant- and microbial-based approaches. This study presents the first report on the biosynthesis of BMNPs using endophytic fungi isolated from medicinal plants. The fungal-synthesized BMNPs demonstrated significant antibacterial and antitumor activities while maintaining excellent biocompatibility. Notably, this biological synthesis method offers distinct advantages over conventional approaches, including environmental sustainability and cost-effectiveness. These findings establish medicinal plant endophytic fungi as a novel and valuable biological resource for producing functional BMNPs with potential biomedical applications.

The easy availability and rapid proliferation of microorganisms make them a highly promising strategy for the biosynthesis of NPs (Rahman et al., 2019). During synthesis, UV-visible spectroscopic analysis of the solution of Ag-CuO NPs revealed the presence of a distinct absorption peak at 467 nm, accompanied by an observation of the color change. Previous studies found that the absorption peaks of Ag–Cu NPs were located within the range of 400–500 nm (Manikandan et al., 2023; Sharma et al., 2021) and the absorption peak of Ag-CuO NPs synthesized by ZMS36 was also in this range. The Ag-CuO NPs were irregularly spherical. The size distribution of the NPs ranged from 11.11 to 69.10 nm, with an average size of 36.63 nm. In previous studies, Ag-CuO NPs synthesized by plant and fungal-mediated methods were also mainly spherical, with particle sizes ranging from 12 to 68 nm (Ameen, 2022; Javid-Naderi et al., 2025; Parvathiraja and Shailajha, 2021; Prashanth et al., 2022). Therefore, our results were consistent with these previous results. The TEM image showed the presence of Ag lattice regions, Cu lattice regions and Ag–Cu alloy regions in the biosynthesized Ag-CuO NPs, which was similar to the Ag–Cu NPs synthesized by Zhu et al. (2021). Fungal-mediated biosynthesis of NPs involves the secretion of various biomolecules, including oxidoreductases and organic acids, which catalyze the reduction of metal ions to their metallic forms (Šebesta et al., 2022; Gudikandula et al., 2017). The fungal-derived proteins and other organic compounds can serve as effective stabilizers by forming capping layers on the surfaces of NPs (Metuku et al., 2014; Roy et al., 2012). Based on the FTIR spectroscopy results, it can be inferred that secondary metabolites such as proteins, polysaccharides, and organic acids, secreted by the endophytic *C. rosea* ZMS36, may serve as reducing and capping agents, thereby facilitating the formation of Ag–CuO NPs.

MRSA continues to be a major public health problem worldwide and presents a therapeutic challenge due to the limited and expensive antibacterial drugs available for treatment (Okwu et al., 2019). The Ag-CuO NPs have been reported to possess good antimicrobial, antitumor, and antioxidant activity (Ameen, 2022; Javid-Naderi et al., 2025; Parvathiraja and Shailajha, 2021; Prashanth et al., 2022). However, no studies have been reported on their activity against MRSA. In this study, Ag-CuO NPs synthesized by strain ZMS36 showed good efficacy in inhibiting the growth of MRSA. The anti-MRSA activity of Ag-CuO NPs can be primarily ascribed to multiple mechanisms. These include altering cell membrane permeability, generating reactive oxygen species (ROS), and disrupting the structural integrity of both the cell wall and membrane (Essghaier et al., 2022; Jalal et al., 2018; Zhou et al., 2021). Moreover, our biosynthesized BMNPs exhibited greater MRSA inhibition activity than the monometallic NPs (AgNPs or CuONPs) in previous studies (Ansari et al., 2015; Cherian et al., 2020; Hamida et al., 2020). This may be attributed to the synergistic effect of the two metals, which enhance the antimicrobial properties of BMNPs (Medina-Cruz et al., 2020). Compared to individual use, the combination of Ag-CuO NPs and vancomycin exhibited significantly enhanced antibacterial efficacy. Similar biological effects have been previously reported, where the combination of ampicillin with Ag-Cu NPs has been shown to enhance the antibacterial effect against *S. pneumoniae* and *P. aeruginosa* (Mujeeb et al., 2020). The observed synergistic antibacterial effect can be attributed to the combined mechanisms of NPs-mediated inhibition of bacterial efflux pumps and biofilm disruption, as well as severe structural damage to bacterial cell walls, which facilitates enhanced NPs penetration (Agreles et al., 2022). This structural compromise facilitates the accumulation of MNPs within the cells, leading to enhanced ROS generation. The combined action of Ag-CuO NPs and antibiotics ultimately restores susceptibility in antibiotic-resistant pathogens and significantly enhances bactericidal efficacy through these complementary pathways of action (Zhao et al., 2023). Additionally, the expression of the MRSA resistance gene (*mecA*) was inhibited by the Ag-CuO NPs. Penicillin-binding protein 2a (PBP2a), encoded by the *mecA* gene, is the primary factor responsible for MRSA's resistance to all β-lactam antimicrobials (Goh et al., 2015). Similarly, Nijil et al. (2024) demonstrated that AgNPs are effective in reducing the expression of the *mecA* gene.

The Ag-CuO NPs demonstrated potent antibacterial activity against a range of bacterial pathogens, including *P. aeruginosa, S. aureus, E. coli, S. epidermidis, S. typhimurium, S. dysenteriae*. The antibacterial activity of Ag-CuO NPs can be attributed to multiple mechanisms, such as altering cell membrane permeability, generating and accumulating ROS, disrupting the structural integrity of the cell and eventually causing the leakage of intracellular contents (Essghaier et al., 2022; Jalal et al., 2018; Zhou et al., 2021). According to the MIC results of this study, the NPs synthesized from endophytic fungi were more effective at inhibiting bacteria than those synthesized by the existing methods. For example, the inhibitory effect of chemically synthesized Ag-Cu NPs and plant-synthesized Ag-Cu NPs and Ag-CuO NPs on *P. aeruginosa, S. aureus* was found to be significantly less potent than the biosynthesized Ag-CuO NPs in this study (Zhu et al., 2021; Mujeeb et al., 2020). The results may be attributable to the binding of bioactive molecules secreted by the endophytic fungus ZMS36 to the biosynthesized Ag-CuO NPs. Given that endophytic fungi produce a diverse array of natural bioactive metabolites that are identical to those of their host plants (Gupta et al., 2020), it is plausible that the bioactive molecules attached to Ag-CuO NPs may exhibit comparable antimicrobial pharmacological effects to those observed in the alcohols and phenols produced by the medicinal plant *A. asphodeloides* (Liu et al., 2023). The results of the FTIR spectroscopy also confirmed the presence of the corresponding substances on the Ag-CuO NPs.

Cancer remains a major global health challenge, particularly cervical and lung carcinomas. However, many traditional therapies approaches have inherent limitations, including variable efficacy across patients and toxic side effects such as anemia, organ damage, hair loss and vomiting (Ramirez et al., 2009). In recent years, nanomaterials have been employed in cancer therapy to address issues of toxicity and enhance drug delivery efficacy (Cheng et al., 2021). In this study, Ag-CuO NPs exhibited significant antiproliferative activity against three different cancer cell lines (A549 cells, PDSF cells, and HeLa cells). It may be due to the elevated intracellular ROS production in tumor cells by inducing oxidative stress. This in turn led to mitochondrial membrane damage and cell cycle arrest, ultimately promoting apoptosis and necrosis in cancer cells (Manikandan et al., 2023; Al-Sheddi et al., 2018). Ag-CuO NPs also could significantly inhibit the migration of HeLa cells and angiogenesis in chick embryos, indicating the potential to inhibit tumor growth and metastasis. The ability of Ag-CuO NPs to differentiate between cancer cells and healthy cells offers a significant advantage of nanotechnology in cancer treatment (Gmeiner and Ghosh, 2014). In addition, Ag-CuO NPs in this study exhibited better antiproliferative activity against HeLa cells than the biosynthesized Ag-CuO NPs by plants (Javid-Naderi et al., 2025). Therefore, the biosynthesized Ag-CuO NPs using endophytic fungi possess great promise for biomedical applications in cancer treatment.

The biosynthesized Ag-CuO NPs in our study exhibited low cytotoxicity toward HaCat cells, providing robust evidence for their safe use. Biosynthesized AgNPs by *Panax ginseng* fresh leaves were found to be non-toxic to HaCaT cell lines at a concentration of 10 μg/mL (Singh et al., 2017). Red blood cells play crucial roles in the human body. Hemolysis, or the rupture of red blood cells, results in the release of hemoglobin, which may lead to the development of anemia, nephrotoxicity and pulmonary hypertension (Rother et al., 2005). Our results indicated that the hemolytic activity of the Ag-CuO NPs was relatively low, which was consistent with the results of other similar studies (Kamli et al., 2021; Singh et al., 2017). The apparent low cytotoxicity of the Ag-Cu NPs against erythrocytes was attributed to the specific modification process involved in their synthesis. Although the evidence suggested that Ag-CuO NPs can be safely utilized at low concentrations over short exposure periods, the prolonged exposure may induce adverse pathological effects, including the development of chronic interstitial pneumonitis and renal accumulation potentially resulting in acute tubular necrosis. The cytotoxic effects of accumulated MNPs in the body may be mediated through multiple pathways, including disrupting the structural integrity of cells, the induction of intracellular inflammatory responses and the generation of ROS. This oxidative stress cascade can subsequently cause DNA damage, ultimately resulting in cellular and tissue injury (Cheng et al., 2021; Xu et al., 2020). Therefore, the long-term biosafety of the Ag-CuO NPs requires further investigation in future researches.

## 5 Conclusion

This study presents, for the first time, an efficient and environmentally friendly synthesis of BMNPs utilizing endophytic fungi isolated from the medicinal plant *A. asphodeloides* to produce Ag-CuO NPs. Characterization analyses revealed that the Ag-CuO NPs were spherical in shape, with an average diameter of 36.63 nm, and were stabilized by a natural coating of bioactive macromolecules secreted by the fungal strain. The synthesized Ag-CuO NPs demonstrated good broad-spectrum antibacterial activity against seven clinically relevant bacterial strains and exhibited prominent dose-dependent anticancer effects against three human tumor cell lines. Notably, the Ag-CuO NPs exhibited low cytotoxicity and demonstrated excellent biocompatibility, highlighting their potential as multifunctional therapeutic agents for both antimicrobial and anticancer applications. These results underscore the potential of medicinal plant endophytic fungi as a sustainable and efficient biological resource for the synthesis of BMNPs. However, it is essential to recognize that the particle size and stability of BMNPs significantly impact their biological activity and therapeutic efficacy. Thus, future research should focus on optimizing synthesis parameters (metal ratios) to produce Ag-CuO NPs with smaller sizes and improved long-term stability. Additionally, investigating the *in vivo* antibacterial efficacy and biosafety of Ag-CuO NPs will be crucial next steps in advancing this research and ensuring their safe and effective application in clinical settings.

## Data Availability

The datasets presented in this study can be found in online repositories. The names of the repository/repositories and accession number(s) can be found below: https://www.ncbi.nlm.nih.gov/nuccore/PP998458.1/.

## References

[B1] Abdelkader D. H. Negm W. A. Elekhnawy E. Eliwa D. Aldosari B. N. Almurshedi A. S.. (2022). Zinc oxide nanoparticles as potential delivery carrier: green synthesis by *Aspergillus niger* endophytic fungus, characterization, and *in vitro*/*in vivo* antibacterial activity. Pharmaceuticals 15:1057. 10.3390/ph15091057 36145278 PMC9500724

[B2] Abdelraheem W. M. Khairy R. M. Zaki A. I. Zaki S. H. (2021). Effect of ZnO nanoparticles on methicillin, vancomycin, linezolid resistance and biofilm formation in *Staphylococcus aureus* isolates. Ann. Clin. Microbiol. Antimicrob. 20:54. 10.1186/s12941-021-00459-2 34419054 PMC8379777

[B3] Abdillah M. Nazazillah N. K. Agustina E. (2017). Identification of active substance in ajwa date (*Phoenix dactylvera* L.) fruit flesh methanol extract. Biotropic 1, 32–39. 10.29080/biotropic.2017.1.1.23-31

[B4] Agreles M. A. A. Cavalcanti I. D. L. Cavalcanti I. M. F. (2022). Synergism between metallic nanoparticles and antibiotics. Appl. Microbiol. Biotechnol. 106, 3973–3984. 10.1007/s00253-022-12001-1 35670851

[B5] Alghuthaymi M. A. Almoammar H. Rai M. Said-Galiev E. Abd-Elsalam K. A. (2015). Myconanoparticles: synthesis and their role in phytopathogens management. Biotechnol. Biotechnol. Equip. 29, 221–236. 10.1080/13102818.2015.1008194 26019636 PMC4433920

[B6] Al-Haddad J. Alzaabi F. Pal P. Rambabu K. Banat F. (2019). Green synthesis of bimetallic copper–silver nanoparticles and their application in catalytic and antibacterial activities. Clean Technol. Environ. Policy 22, 269–277. 10.1007/s10098-019-01765-2

[B7] Al-Sheddi E. S. Farshori N. N. Al-Oqail M. M. Al-Massarani S. M. Saquib Q. Wahab R.. (2018). Anticancer potential of green synthesized silver nanoparticles using extract of Nepeta deflersiana against human cervical cancer cells (HeLA). Bioinorg. Chem. Appl. 2018:9390784. 10.1155/2018/9390784 30515193 PMC6236914

[B8] Ameen F. (2022). Optimization of the synthesis of fungus-mediated bi-metallic Ag-Cu nanoparticles. Appl. Sci. 12:1384. 10.3390/app12031384

[B9] Amina M. Al Musayeib N. M. Alarfaj N. A. El-Tohamy M. F. Al-Hamoud G. A. (2020). Antibacterial and immunomodulatory potentials of biosynthesized Ag, Au, Ag-Au bimetallic alloy nanoparticles using the asparagus racemosus root extract. Nanomaterials 10:2453. 10.3390/nano10122453 33302432 PMC7762544

[B10] Anil Kumar S. Abyaneh M. K. Gosavi S. Kulkarni S. K. Pasricha R. Ahmad A.. (2007). Nitrate reductase-mediated synthesis of silver nanoparticles from AgNO_3_. Biotechnol. Lett. 29, 439–445. 10.1007/s10529-006-9256-7 17237973

[B11] Ansari M. A. Khan H. M. Khan A. Cameotra S. Alzohairy M. (2015). Anti-biofilm efficacy of silver nanoparticles against MRSA and MRSE isolated from wounds in a tertiary care hospital. Indian J. Med. Microbiol. 33, 101–109. 10.4103/0255-0857.148402 25560011

[B12] Arora N. Thangavelu K. Karanikolos G. N. (2020). Bimetallic nanoparticles for antimicrobial applications. Front. Chem. 8:412. 10.3389/fchem.2020.00412 32671014 PMC7326054

[B13] Cheng Z. Li M. Dey R. Chen Y. (2021). Nanomaterials for cancer therapy: current progress and perspectives. J. Hematol. Oncol. 14:85. 10.1186/s13045-021-01096-0 34059100 PMC8165984

[B14] Cherian T. Ali K. Saquib Q. Faisal M. Wahab R. Musarrat J.. (2020). Cymbopogon citratus functionalized green synthesis of CuO-nanoparticles: novel prospects as antibacterial and antibiofilm agents. Biomolecules 10:169. 10.3390/biom10020169 31979040 PMC7072505

[B15] Chew Y. L. Mahadi A. M. Wong K. M. Goh J. K. (2018). Anti-methicillin-resistance *Staphylococcus aureus* (MRSA) compounds from Bauhinia kockiana Korth. And their mechanism of antibacterial activity. BMC Complement. Alternat. Med. 18, 1–9. 10.1186/s12906-018-2137-5 29463252 PMC5819667

[B16] CLSI (2024). Performance Standards for Antimicrobial Susceptibility Testing, 34th Edn. CLSI supplement M100. Wayne, PA: Clinical and Laboratory Standards Institute.

[B17] Deepa K. Panda T. (2014). Synthesis of gold nanoparticles from different cellular fractions of *Fusarium oxysporum*. J. Nanosci. Nanotechnol. 14, 3455–3463. 10.1166/jnn.2014.8247 24734569

[B18] Deng Z. Zhang R. Shi Y. La H. Tan H. Cao L. (2014). Characterization of Cd-, Pb-, Zn-resistant endophytic *Lasiodiplodia* sp. MXSF31 from metal accumulating Portulaca oleracea and its potential in promoting the growth of rape in metal-contaminated soils. Environ. Sci. Pollut. Res. 21, 2346–2357. 10.1007/s11356-013-2163-2 24062066

[B19] Devi L. S. Bareh D. A. Joshi S. (2014). Studies on biosynthesis of antimicrobial silver nanoparticles using endophytic fungi isolated from the ethno-medicinal plant *Gloriosa superba* L. Proc. Natl. Acad. Sci. India Sec. B Biol. Sci. 84, 1091–1099. 10.1007/s40011-013-0185-7

[B20] Dobrucka R. Dlugaszewska J. (2018). Antimicrobial activity of the biogenically synthesized core-shell Cu@Pt nanoparticles. Saudi Pharm. J. 26, 643–650. 10.1016/j.jsps.2018.02.028 29991908 PMC6035316

[B21] Durán N. Marcato P. D. Alves O. L. De Souza G. I. Esposito E. (2005). Mechanistic aspects of biosynthesis of silver nanoparticles by several *Fusarium oxysporum* strains. J. Nanobiotechnol. 3, 1–7. 10.1186/1477-3155-3-8 16014167 PMC1180851

[B22] Essghaier B. Toukabri N. Dridi R. Hannachi H. Limam I. Mottola F.. (2022). First report of the biosynthesis and characterization of silver nanoparticles using *Scabiosa atropurpurea* subsp. maritima fruit extracts and their antioxidant, antimicrobial and cytotoxic properties. Nanomaterials 12:1585. 10.3390/nano12091585 35564294 PMC9104986

[B23] Fan X. Yahia L. H. Sacher E. (2021). Antimicrobial properties of the Ag, Cu nanoparticle system. Biology 10:137. 10.3390/biology10020137 33578705 PMC7916421

[B24] Gmeiner W. H. Ghosh S. (2014). Nanotechnology for cancer treatment. Nanotechnol. Rev. 3, 111–122. 10.1515/ntrev-2013-0013 26082884 PMC4465796

[B25] Goh S. Loeffler A. Lloyd D. H. Nair S. P. Good L. (2015). Oxacillin sensitization of methicillin-resistant Staphylococcus aureus and methicillin-resistant Staphylococcus pseudintermedius by antisense peptide nucleic acids *in vitro*. BMC Microbiol. 15:262. 10.1186/s12866-015-0599-x 26560174 PMC4642645

[B26] Gudikandula K. Vadapally P. Charya M. S. (2017). Biogenic synthesis of silver nanoparticles from white rot fungi: their characterization and antibacterial studies. OpenNano 2, 64–78. 10.1016/j.onano.2017.07.002

[B27] Gupta S. Chaturvedi P. Kulkarni M. G. Van Staden J. (2020). A critical review on exploiting the pharmaceutical potential of plant endophytic fungi. Biotechnol. Adv. 39:107462. 10.1016/j.biotechadv.2019.107462 31669137

[B28] Hamida R. S. Ali M. A. Goda D. A. Khalil M. I. Al-Zaban M. I. (2020). Novel biogenic silver nanoparticle-induced reactive oxygen species inhibit the biofilm formation and virulence activities of methicillin-resistant *Staphylococcus aureus* (MRSA) strain. Front. Bioeng. Biotechnol. 8:433. 10.3389/fbioe.2020.00433 32548095 PMC7270459

[B29] Hosseini M. R. Sarvi M. N. (2015). Recent achievements in the microbial synthesis of semiconductor metal sulfide nanoparticles. Mater. Sci. Semiconduct. Process. 40, 293–301. 10.1016/j.mssp.2015.06.003

[B30] Ibrahim S. A. Fayed E. A. Rizk H. F. Desouky S. E. Ragab A. (2021). Hydrazonoyl bromide precursors as DHFR inhibitors for the synthesis of bis-thiazolyl pyrazole derivatives; antimicrobial activities, antibiofilm, and drug combination studies against MRSA. Bioorg. Chem. 116:105339. 10.1016/j.bioorg.2021.105339 34530234

[B31] Jalal M. Ansari M. A. Alzohairy M. A. Ali S. G. Khan H. M. Almatroudi A.. (2018). Biosynthesis of silver nanoparticles from oropharyngeal *Candida glabrata* isolates and their antimicrobial activity against clinical strains of bacteria and fungi. Nanomaterials 8:586. 10.3390/nano8080586 30071582 PMC6116273

[B32] Jamil N. Saad Ali H. M. Yasir M. Hamza M. Sagheer M. Ahmed T.. (2024). Biosynthesized metallic and bimetallic nanoparticles as effective biocides for plant protection: plausible mechanisms and challenges. J. Chem. 2024. 10.1155/2024/3328223

[B33] Javid-Naderi M. J. Sabouri Z. Jalili A. Zarrinfar H. Sammak S. Darroudi M.. (2025). Green synthesis and characterization of Ag/CuO nanoparticles: exploring their antifungal, antimicrobial, and cytotoxic properties. Environ. Technol. Innov. 38:104147. 10.1016/j.eti.2025.104147

[B34] Jiang X. Fan X. Xu W. Zhang R. Wu G. (2020). Biosynthesis of bimetallic Au-Ag nanoparticles using *Escherichia coli* and its biomedical applications. ACS Biomater. Sci. Eng. 6, 680–689. 10.1021/acsbiomaterials.9b01297 33463224

[B35] Kali A. Srirangaraj S. Charles M. P. (2014). A modified fungal slide culture technique. Indian J. Pathol. Microbiol. 57, 356–357. 10.4103/0377-4929.134756 24943797

[B36] Kamli M. R. Malik M. A. Lone S. A. Sabir J. S. M. Mattar E. H. Ahmad A.. (2021). Beta vulgaris assisted fabrication of novel Ag-Cu bimetallic nanoparticles for growth inhibition and virulence in *Candida albicans*. Pharmaceutics 13:1957. 10.3390/pharmaceutics13111957 34834372 PMC8621205

[B37] Kot B. Sytykiewicz H. Sprawka I. (2018). Expression of the biofilm-associated genes in methicillin-resistant *Staphylococcus aureus* in biofilm and planktonic conditions. Int. J. Mol. Sci. 19:3487. 10.3390/ijms19113487 30404183 PMC6274806

[B38] Kumari M. Sharma N. Manchanda R. Gupta N. Syed A. Bahkali A. H.. (2021). PGMD/curcumin nanoparticles for the treatment of breast cancer. Sci. Rep. 11:3824. 10.1038/s41598-021-81701-x 33589661 PMC7884397

[B39] Kumari M. M. Jacob J. Philip D. (2015). Green synthesis and applications of Au–Ag bimetallic nanoparticles. Spectrochim. Acta A Mol. Biomol. Spectrosc. 137, 185–192. 10.1016/j.saa.2014.08.079 25218228

[B40] Letchumanan D. Sok S. P. Ibrahim S. Nagoor N. H. Arshad N. M. (2021). Plant-based biosynthesis of copper/copper oxide nanoparticles: an update on their applications in biomedicine, mechanisms, and toxicity. Biomolecules 11:564. 10.3390/biom11040564 33921379 PMC8069291

[B41] Li J. Xie S. Gao Q. Deng Z. (2024). Evaluation of the potential of endophytic *Trichoderma* sp. isolated from medicinal plant *Ampelopsis japonica* against MRSA and bioassay-guided separation of the anti-MRSA compound. Brazil. J. Microbiol. 55, 543–556. 10.1007/s42770-024-01250-z 38261262 PMC10920522

[B42] Liu C. Cong Z. Wang S. Zhang X. Song H. Xu T.. (2023). review of the botany, ethnopharmacology, phytochemistry, pharmacology, toxicology and quality of *Anemarrhena asphodeloides* Bunge. *J. Ethnopharmacol*. 302(Pt A):115857. 10.1016/j.jep.2022.115857 36330891

[B43] Lodi R. S. Dong X. Wang X. Han Y. Liang X. Peng C.. (2025). Current research on the medical importance of *Trametes* species. Fungal Biol. Rev. 51:100413. 10.1016/j.fbr.2025.100413

[B44] Loza K. Heggen M. Epple M. (2020). Synthesis, structure, properties, and applications of bimetallic nanoparticles of noble metals. Adv. Funct. Mater. 30:1909260. 10.1002/adfm.201909260

[B45] Luo P. Feng X. Liu S. Jiang Y. (2024). Traditional uses, phytochemistry, pharmacology and toxicology of *Ruta graveolens* L.: a critical review and future perspectives. Drug Des. Dev. Ther. 18, 6459–6485. 10.2147/DDDT.S494417 39758226 PMC11697671

[B46] Ma H. Duan X. Xu W. Ma G. Ma W. Qi H. (2022). Root Rot of *Angelica sinensis* caused by *Clonostachys rosea* and *Fusarium acuminatum* in China. Plant Dis. 106:2264. 10.1094/PDIS-12-21-2665-PDN35077224

[B47] Mani V. M. Kalaivani S. Sabarathinam S. Vasuki M. Soundari A. Ayyappa Das M. P.. (2021). Copper oxide nanoparticles synthesized from an endophytic fungus *Aspergillus terreus*: bioactivity and anti-cancer evaluations. Environ. Res. 201:111502. 10.1016/j.envres.2021.111502 34214561

[B48] Manikandan D. B. Arumugam M. Sridhar A. Perumalsamy B. Ramasamy T. (2023). Sustainable fabrication of hybrid silver-copper nanocomposites (Ag-CuO NCs) using *Ocimum americanum* L. as an effective regime against antibacterial, anticancer, photocatalytic dye degradation and microalgae toxicity. Environ. Res. 228:115867. 10.1016/j.envres.2023.115867 37044164

[B49] Medina-Cruz D. Saleh B. Vernet-Crua A. Nieto-Argüello A. Lomelí-Marroquín D. Vélez-Escamilla L. Y.. (2020). “Bimetallic nanoparticles for biomedical applications: a review,” in Racing for the Surface: Antimicrobial and Interface Tissue Engineering, eds. B. Li, T. F. Moriarty, T. Webster, and M. Xing (Cham: Springer International Publishing), 397–434. 10.1007/978-3-030-34471-9_16

[B50] Merugu R. Gothalwal R. Kaushik Deshpande P. De Mandal S. Padala G. Latha Chitturi K.. (2021). Synthesis of Ag/Cu and Cu/Zn bimetallic nanoparticles using toddy palm: investigations of their antitumor, antioxidant and antibacterial activities. Mater. Today Proc. 44, 99–105. 10.1016/j.matpr.2020.08.027

[B51] Metuku R. P. Pabba S. Burra S. Hima Bindu N. S. Gudikandula K. Singara Charya M. A.. (2014). Biosynthesis of silver nanoparticles from Schizophyllum radiatum HE 863742.1: their characterization and antimicrobial activity. 3 Biotech. 4, 227–234. 10.1007/s13205-013-0138-0 28324427 PMC4026449

[B52] Mistry H. Thakor R. Patil C. Trivedi J. Bariya H. (2021). Biogenically proficient synthesis and characterization of silver nanoparticles employing marine procured fungi *Aspergillus brunneoviolaceus* along with their antibacterial and antioxidative potency. Biotechnol. Lett. 43, 307–316. 10.1007/s10529-020-03008-7 32944816

[B53] Mohamed N. H. Ismail M. A. Abdel-Mageed W. M. Mohamed Shoreit A. A. (2019). Antimicrobial activity of green silver nanoparticles from endophytic fungi isolated from *Calotropis procera* (Ait) latex. Microbiology 165, 967–975. 10.1099/mic.0.000832 31309923

[B54] Mujeeb A. A. Khan N. A. Jamal F. Badre Alam K. F. Saeed H. Kazmi S.. (2020). Olax scandens mediated biogenic synthesis of Ag-Cu nanocomposites: potential against inhibition of drug-resistant microbes. Front. Chem. 8:103. 10.3389/fchem.2020.00103 32185160 PMC7058794

[B55] Nijil S. Bhat S. G. Kedla A. Thomas M. R. Kini S. (2024). A silver lining in MRSA treatment: the synergistic action of poloxamer-stabilized silver nanoparticles and methicillin against antimicrobial resistance. Microb. Pathog. 197:107087. 10.1016/j.micpath.2024.107087 39481693

[B56] Okwu M. U. Olley M. Akpoka A. O. Izevbuwa O. E. (2019). Methicillin-resistant *Staphylococcus aureus* (MRSA) and anti-MRSA activities of extracts of some medicinal plants: a brief review. AIMS Microbiol. 5:117. 10.3934/microbiol.2019.2.117 31384707 PMC6642907

[B57] Parvathiraja C. Shailajha S. (2021). Bioproduction of CuO and Ag/CuO heterogeneous photocatalysis-photocatalytic dye degradation and biological activities. Appl. Nanosci. 11, 1411–1425. 10.1007/s13204-021-01743-5

[B58] Peng C. Wang Q. Xu W. Wang X. Zheng Q. Liang X.. (2024). A bifunctional endolytic alginate lyase with two different lyase catalytic domains from *Vibrio* sp. H204. Front. Microbiol. 15:1509599. 10.3389/fmicb.2024.1509599 39735187 PMC11671496

[B59] Pesic M. Podolski-Renic A. Stojkovic S. Matovic B. Zmejkoski D. Kojic V.. (2015). Anti-cancer effects of cerium oxide nanoparticles and its intracellular redox activity. Chem. Biol. Interact. 232, 85–93. 10.1016/j.cbi.2015.03.013 25813935

[B60] Prabakaran S. Rajan M. (2021). Biosynthesis of nanoparticles and their roles in numerous areas. Comprehens. Anal. Chem. 94, 1–47. 10.1016/bs.coac.2021.02.001

[B61] Prashanth G. K. Sathyananda H. M. Prashanth P. A. Gadewar M. Mutthuraju M. Prabhu S. R. B.. (2022). Controlled synthesis of Ag/CuO nanocomposites: evaluation of their antimycobacterial, antioxidant, and anticancer activities. Appl. Phys. A 128:614. 10.1007/s00339-022-05748-x

[B62] Rahman S. Rahman L. Khalil A. T. Ali N. Zia D. Ali M.. (2019). Endophyte-mediated synthesis of silver nanoparticles and their biological applications. Appl. Microbiol. Biotechnol. 103, 2551–2569. 10.1007/s00253-019-09661-x 30721330

[B63] Ramirez L. Y. Huestis S. E. Yap T. Y. Zyzanski S. Drotar D. Kodish E.. (2009). Potential chemotherapy side effects: what do oncologists tell parents? Pediatric Blood Cancer 52, 497–502. 10.1002/pbc.21835 19101994 PMC2643320

[B64] Riaz T. Mughal P. Shahzadi T. Shahid S. Abbasi M. A. (2020). Green synthesis of silver nickel bimetallic nanoparticles using plant extract of *Salvadora persica* and evaluation of their various biological activities. Mater. Res. Express 6, 1250–1253. 10.1088/2053-1591/ab74fc

[B65] Rosbero T. M. S. Camacho D. H. (2017). Green preparation and characterization of tentacle-like silver/copper nanoparticles for catalytic degradation of toxic chlorpyrifos in water. J. Environ. Chem. Eng. 5, 2524–2532. 10.1016/j.jece.2017.05.009

[B66] Rother R. P. Bell L. Hillmen P. Gladwin M. T. (2005). The clinical sequelae of intravascular hemolysis and extracellular plasma hemoglobin: a novel mechanism of human disease. JAMA 293, 1653–1662. 10.1001/jama.293.13.1653 15811985

[B67] Roy M. Mukherjee P. Mandal B. P. Sharma R. K. Tyagi A. K. Kale S. P.. (2012). Biomimetic synthesis of nanocrystalline silver sol using cysteine: stability aspects and antibacterial activities. RSC Adv. 2, 6496–6503. 10.1039/c2ra00785a

[B68] Šebesta M. Vojtková H. Cyprichová V. Ingle A. P. Urík M. Kolenčík M.. (2022). Mycosynthesis of metal-containing nanoparticles—fungal metal resistance and mechanisms of synthesis. Int. J. Mol. Sci. 23:14084. 10.3390/ijms232214084 36430561 PMC9696665

[B69] Sengupta D. Mondal B. Mukherjee K. (2015). Visible light absorption and photo-sensitizing properties of spinach leaves and beetroot extracted natural dyes. Spectrochim. Acta A Mol. Biomol. Spectrosc. 148, 85–92. 10.1016/j.saa.2015.03.120 25875029

[B70] Sharma D. Kanchi S. Bisetty K. (2019). Biogenic synthesis of nanoparticles: a review. Arab. J. Chem. 12, 3576–3600. 10.1016/j.arabjc.2015.11.002

[B71] Sharma D. Ledwani L. Kumar N. Mehrotra T. Pervaiz N. Kumar R.. (2021). An investigation of physicochemical and biological properties of rheum emodi-mediated bimetallic Ag–Cu nanoparticles. Arab. J. Sci. Eng. 46, 275–285. 10.1007/s13369-020-04641-0

[B72] Shkryl Y. Rusapetova T. Yugay Y. Egorova A. Silant'ev V. Grigorchuk V.. (2021). Biosynthesis and cytotoxic properties of Ag, Au, and bimetallic nanoparticles synthesized using Lithospermum erythrorhizon callus culture extract. Int. J. Mol. Sci. 22:9305. 10.3390/ijms22179305 34502210 PMC8431615

[B73] Singh P. Kim Y.-J. Zhang D. Yang D.-C. (2016). Biological synthesis of nanoparticles from plants and microorganisms. Trends Biotechnol. 34, 588–599. 10.1016/j.tibtech.2016.02.006 26944794

[B74] Singh P. Singh H. Ahn S. Castro-Aceituno V. Jiménez Z. Simu S. Y.. (2017). Pharmacological importance, characterization and applications of gold and silver nanoparticles synthesized by *Panax ginseng* fresh leaves. Artificial Cells Nanomed. Biotechnol. 45, 1415–1424. 10.1080/21691401.2016.1243547 27855495

[B75] Tian J. Wei X. Zhang W. Xu A. (2020). Effects of selenium nanoparticles combined with radiotherapy on lung cancer cells. Front. Bioeng. Biotechnol. 8:598997. 10.3389/fbioe.2020.598997 33304892 PMC7701302

[B76] Velsankar K. Aswin Kumar R. M. Preethi R. Muthulakshmi V. Sudhahar S. (2020). Green synthesis of CuO nanoparticles via *Allium sativum* extract and its characterizations on antimicrobial, antioxidant, antilarvicidal activities. J. Environ. Chem. Eng. 8:104123. 10.1016/j.jece.2020.104123

[B77] Wang K. Nicholaou M. (2017). Suppression of antimicrobial resistance in MRSA using CRISPR-dCas9. Am. Soc. Clin. Lab. Sci. 30, 207–213. 10.29074/ascls.30.4.207

[B78] Xu L. Wang Y.-Y. Huang J. Chen C.-Y. Wang Z.-X.. (2020). Silver nanoparticles: synthesis, medical applications and biosafety. Theranostics 10, 8996–9031. 10.7150/thno.45413 32802176 PMC7415816

[B79] Zawadzka K. Felczak A. Nowak M. Kowalczyk A. Piwoński I. Lisowska K.. (2021). Antimicrobial activity and toxicological risk assessment of silver nanoparticles synthesized using an eco-friendly method with *Gloeophyllum striatum*. J. Hazardous Mater. 418:126316. 10.1016/j.jhazmat.2021.126316 34118550

[B80] Zeng G. Wu Z. Cao W. Wang Y. Deng X. Zhou Y.. (2020). Identification of anti-nociceptive constituents from the pollen of *Typha angustifolia* L. using effect-directed fractionation. Nat. Product Res. 34, 1041–1045. 10.1080/14786419.2018.1539979 30580603

[B81] Zhang H. Jin M. Liu H. Wang J. Kim M. J. Yang D.. (2011). Facile synthesis of Pd–Pt alloy nanocages and their enhanced performance for preferential oxidation of CO in excess hydrogen. ACS Nano 5, 8212–8222. 10.1021/nn202896q 21888409

[B82] Zhao X. Liu S. Jiang X. (2023). Leveraging bimetallic nanoparticles to synergistically enhance the efficacy of antibiotics against carbapenem-resistant bacteria. Sci. China Mater. 66, 2885–2892. 10.1007/s40843-022-2459-3

[B83] Zhao Y. Ye C. Liu W. Chen R. Jiang X. (2014). Tuning the composition of AuPt bimetallic nanoparticles for antibacterial application. Angew. Chem. Int. Ed. 53, 8127–8131. 10.1002/anie.201401035 24828967 PMC4320751

[B84] Zhou L. Zhao X. Li M. Lu Y. Ai C. Jiang C.. (2021). Antifungal activity of silver nanoparticles synthesized by iturin against *Candida albicans in vitro* and *in vivo*. Appl. Microbiol. Biotechnol. 105, 3759–3770. 10.1007/s00253-021-11296-w 33900424

[B85] Zhu Y. Zhou F. Hu J. Yang L. Yang D.-Q. Sacher E.. (2021). A facile route to prepare colorless Ag-Cu nanoparticle dispersions with elevated antibacterial effects. Colloids Surf. A Physicochem. Eng. Aspects 626:127116. 10.1016/j.colsurfa.2021.127116

