# Supplementary material for: Biosynthesis of bimetallic silver–copper oxide nanoparticles using endophytic Clonostachys rosea ZMS36 and their biomedical applications
Source: Front Microbiol. 2025 May 9;16:1581486. doi: 10.3389/fmicb.2025.1581486 (PMC12098627; doi:10.3389/fmicb.2025.1581486)

**Figure S1.** Effects of metal salt concentrations (A), reaction time (B) and pH (C) on the sizes of Ag-CuO NPs. ***** represented the failure to synthesize Ag-CuO NPs. Different lowercase letters indicated significant difference in the sizes of Ag-CuO NPs


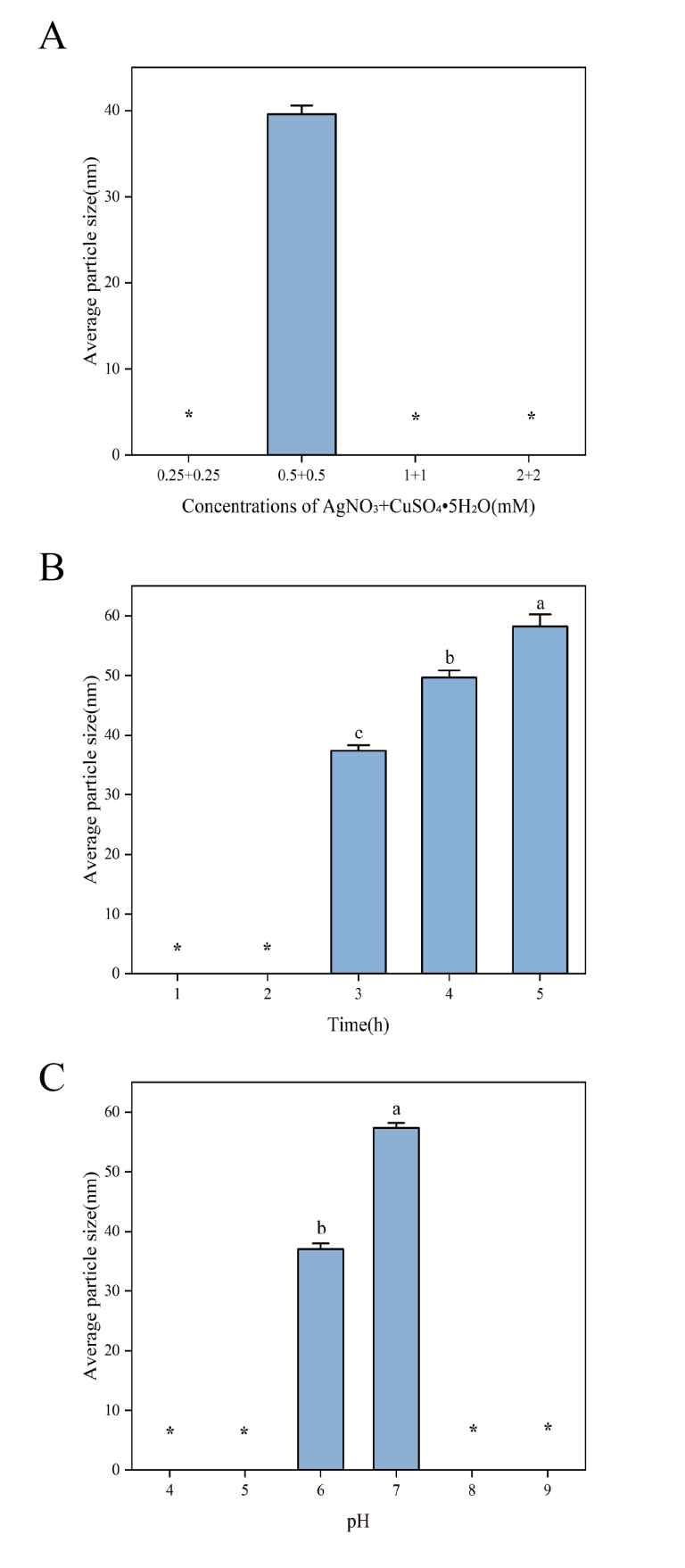

Supplement: Supplementary file 1 [file Supplementary_file_1.docx]
